# Semi-automatic liver segmentation based on probabilistic models and anatomical constraints

**DOI:** 10.1038/s41598-021-85436-7

**Published:** 2021-03-17

**Authors:** Doan Cong Le, Krisana Chinnasarn, Jirapa Chansangrat, Nattawut Keeratibharat, Paramate Horkaew

**Affiliations:** 1 School of Computer Engineering, Institute of Engineering, Suranaree University of Technology, Nakhon Ratchasima, Thailand; 2 Department of Computer Science, Faculty of Informatics, Burapha University, Chon Buri, Thailand; 3 School of Radiology, Institute of Medicine, Suranaree University of Technology, Nakhon Ratchasima, Thailand; 4 School of Surgery, Institute of Medicine, Suranaree University of Technology, Nakhon Ratchasima, Thailand

**Keywords:** Computational models, Computational platforms and environments, Data processing, Image processing, Software, Anatomy, Biomarkers, Gastroenterology, Computer science, Information technology, Translational research

## Abstract

Segmenting a liver and its peripherals from abdominal computed tomography is a crucial step toward computer aided diagnosis and therapeutic intervention. Despite the recent advances in computing methods, faithfully segmenting the liver has remained a challenging task, due to indefinite boundary, intensity inhomogeneity, and anatomical variations across subjects. In this paper, a semi-automatic segmentation method based on multivariable normal distribution of liver tissues and graph-cut sub-division is presented. Although it is not fully automated, the method minimally involves human interactions. Specifically, it consists of three main stages. Firstly, a subject specific probabilistic model was built from an interior patch, surrounding a seed point specified by the user. Secondly, an iterative assignment of pixel labels was applied to gradually update the probabilistic map of the tissues based on spatio-contextual information. Finally, the graph-cut model was optimized to extract the 3D liver from the image. During post-processing, overly segmented nodal regions due to fuzzy tissue separation were removed, maintaining its correct anatomy by using robust bottleneck detection with adjacent contour constraint. The proposed system was implemented and validated on the MICCAI SLIVER07 dataset. The experimental results were benchmarked against the state-of-the-art methods, based on major clinically relevant metrics. Both visual and numerical assessments reported herein indicated that the proposed system could improve the accuracy and reliability of asymptomatic liver segmentation.

## Introduction

**Figure 1:**
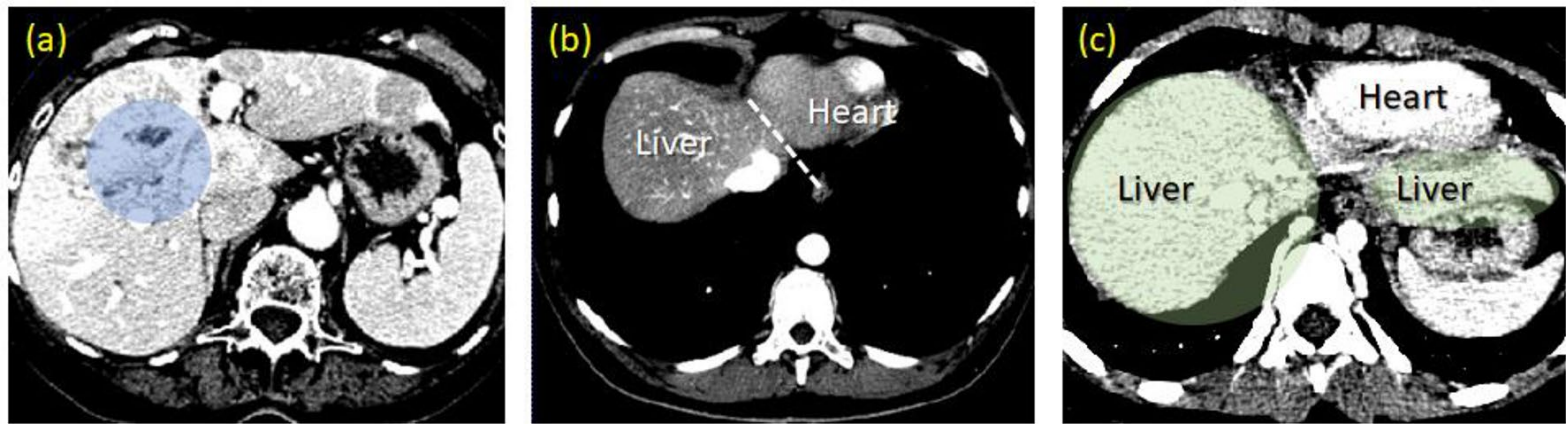
Challenges associated with liver segmentation, i.e., inhomogeneity of intensity in the liver region (**a**), fuzzy separation between liver and heart (**b**) and the multi-segments geometry within single slide (**c**). In addition, these cases exhibit different intensity ranges of liver tissue.

Liver is the largest organ in the abdomen. It functions as a filter, which prevents waste and other toxins being released into the circular system. Because a diseased liver cannot properly maintain its performance, earliest detection of pathological manifestations ensures the effectiveness of treatments, and hence prolonging patient’s life. Moreover, modern developments in therapeutic intervention and surgery have increasingly relied on computerized reconstruction of a subject-specific liver, both for treatment planning and in subsequent proceeding. Computed Tomography (CT) is one of the primary modalities preferred in those platforms. It allows the physician to clearly visualize anatomical structure of this organ as well as pathological evidence. However, prognostic assessment during a treatment requires standardized protocols, which in turn call for quantitating pathological indicators. They usually involve delineation of liver, its peripherals, and lesions (if any) by an experienced and skilled radiologist. The process is known for being laborious, time consuming, and prone to inter- and intra-observer variabilities. Recently, it has been even more so, given increasing resolution of a 3D matrix acquired by a modern CT^1–4^. Consequently, fully- and semi-automatic system has become vital in Computer Aided Diagnosis (CAD). Unlike other imaging protocols, however, CT liver is difficult to be segmented, due to inheriting challenges (Fig. 1).

Since CT element represents tissue as its X-ray absorption, it does inevitably not differentiate well adjacent organs with similar properties, such as kidney, heart, and muscle from the liver. In addition, there is no edge gradient sufficiently strong to be identified as a boundary separating these objects. In fact, inhomogeneity of liver interior is much pronounced than that against non-liver regions, leading to low segmenting accuracy. Disease induced changes and deformity^1,5,6^, motion artefacts, as well as inter-subject variability, also worsen the outcomes. Last but not least, as the liver geometry is very complex but still singly connected, it may appear as separate regions in some slices. Simplifying 3D segmentation to 2D contour tracing is not trivial. Due to these challenges, faithfully segmenting a liver from volumetric CT images thus largely remains an open area of investigation.

It has long been debated whether a fully or semi-automatic method is suitable for a given CAD problem. Previous studies have attempted liver segmenting methods, implemented on various medical systems. It was generally perceived that those with higher degree of user interaction involved outperforms their counterparts, inevitably at a cost of greater time consuming and endeavor required. On the other hand, improving segmenting accuracy of fully automatic liver extraction often relied on supervised machine learning (ML) strategies and expert systems that required model training, and thus large amount of data, which is not always available^3^. Inspired by the dilemma, this paper considered the balance between segmenting accuracy and user interaction, suitable for typical clinical setting with limited domain of experts. It proposes a semi-automatic liver segmentation from 3D CT images. Our contributions are three folds. Firstly, interaction is limited to merely specifying a seed point within a liver by means of straightforward graphic user interface (GUI). Secondly, it ensures the robustness by employing a probabilistic framework in refining preliminary classification. Finally, the correctness of liver anatomy is asserted by using bottle-neck detection and adjacent contour constraints to remove over segmented regions.

This paper is organized as follow: After this, relevant state-of-the-art methods in the field are reviewed. Detailed description of the proposed liver segmentation, consisting of multivariate modeling of pixel intensities, probabilistic framework for fine-tuning the tissue labels, and anatomical-constrained post-processing are then provided. In subsequent sections, experiments and results on standard dataset, including subjective and numerical evaluations, and their discussions are presented. Lastly, concluding remarks on the proposed scheme and its prospects in computerized medical systems are made.

## Related studies

This section focuses on extracting liver from CT imaging. Existing techniques mostly differed in feature selection, amount of user interaction involved, and modeling constraints imposed during the procedure. An overview of recent developments can be found in^7–9^, while detailed evaluations and their benchmarking, based on common dataset, are presented in^3^. However, some of the prominent studies are reviewed here. They can be divided into those using fully and semi-automatic approaches.

### Fully automatic segmentation

Most automated segmentation methods relied on statistical model of a liver shape, either via initialization or as a constraint. However, it was reported that extent to which a model could capture plausible variations found in typical shape space are determined by the number and resolution of training liver instances^3^. In^10,11^, for examples, a set of landmarks built from a dataset was used to fit a deformable model to a liver image. Those works built their models from 20 and 112 livers, using 2500 and 7000 model parameters, respectively. Learning from known instances, Cheng et al.^12^, proposed a combination of active appearance model (AAM), live wire (LW), and graph-cut for learning textual model, recognizing object of interest, and obtaining its final clustering, respectively. Similarly, Li et al.^13^, imposed morphological constraint on an initial boundary for anatomically plausible liver, by means of principal component analysis (PCA). Any excessive variations left in unseen instances was regulated by deformable graph cut. Most recent and rapid development of convolution neural network (CNN) has enabled much efficient delineation of liver boundary. Lu et al.^14^ estimated a liver surface by pixelwise probabilistic model, trained by CNN from 78 CT images. Any variations unrecoverable by CNN were similarly enhanced by using graph-cut. Similar approach was taken by Lu and Hu^15^, but the resultant probabilistic map was used to optimize surface evolution. In this work, the model was learnt from 109 CT images. Considering pathological cases, Li et al.^16^ adopted hybrid (2D and 3D) densely connected UNet (referred to as H-DenseUNet) for segmenting both liver and liver tumor. The 2D DenseUNet was used to extract their features within a slice, while 3D DenseUNet allowed learning of spatial information between consecutive ones. These DenseUNet models were fused and optimized to obtain final liver and tumor segmentation. Despite relatively high scores in its class, these models took 9 h to converge and 30 h in total for training. Once completed, a new instance could be segmented within 30 to 200 s per image.

Addressing some issues found in treating a real patient underwent liver transplant, another work^17^ proposed parallel learning and segmenting liver from abdominal CT angiography (CTA). In this work, CTA images were divided into low and high contrast groups. It made use of knowledge on the anatomy of kidneys, ribs, and livers, in combination with thresholding technique, to remove irrelevant parts and to highlight the region of interest (ROI). K-Means and Multi-Layer Perceptron (MLP) classifiers were subsequently applied to high and low contrast data, respectively, depending on their histogram appearances, based on automatic switching mechanism. Heuristic post processing was finally used to remove over-segments, while remaining errors may be manually corrected. Another more recent study^18^ by Zheng et al., built a liver classifier from twelve textual features, calculated from a gray level co-occurrence matrix (GLCM). In addition, liver position was exploited as contextual information in another classifier. Probabilities computed from these classifiers were integrated into a random-walk model to obtain the final segmentation. In their experiment, 18 slices, each containing the largest liver area of a subject, were used in training, while 2 CT volumes were used for testing. Note that shape information of the liver was not considered.

In the absence of training set, some studies relied only on information extracted from CT images. However, a liver contour, automatically estimated in heterogeneity was unstable. This as well applies to seed point or marker, especially when being placed on pathological region. Marcin^19^ constructed 2D liver contour by combining both left and right-hand side ones, defined by 5 and 3 polylines, respectively. Provided a centroid of an image, a starting point of a contour was first located by comparing its intensity with that of lumbar spine section. Subsequent points were iteratively traced on respective polyline, based on their geometric distance to a current point and its intensity within discretized ranges. A shortcoming of this method was being dependent on the location of lumbar spine and symmetry of an input image. Additionally, directly comparing intensities between points on a polyline was sensitive to imaging noise. Wu et al.^20^ computed maximum intensity projection (MIP) of 3D CT to determine the abdominal region. Threshold and morphology methods were applied to determine the volume of interest (VOI). Finally, linear iterative clustering and graph-cut were utilized to segment the super-voxel liver. Another more generic approach was proposed by Kumar et al.^21^. They applied region growing out of seed points that were automatically selected by thresholding. Lesions were extracted from the resultant liver by means of a modified Fuzzy C-Mean algorithm. Recently, Huang et al.^22^ divided a CT image into subregions by using K-Mean, computed on an initial slice. A contour was then roughly estimated as that enclosing one with the highest number of pixels. Graph-cut with Gaussian parameters and inter-slice gradient being incorporated into region and boundary terms, respectively, were applied to assemble small regions. Vena cava was detached by a rectangular template. Other over-segments were removed, if they were less overlapped with a specified template and their average intensities fell out of a specified range. Interior void due to tumor was discarded by concave filling, except, however, those on boundary.

### Semi-automatic segmentation

The methods in this category require user interaction either on initializing segmentation or imposing constraints. Some examples include^23–25^, in which a level-set was employed with user interaction to an extent to complete the process. More specifically, in^23^, a user was asked to provide an initial liver contour in some slices, while in^24^ seed points on top and bottom parts in every lobe of a liver were needed. Fewer seed points manually initialized on some slices were used in^25^ to define an initial area for fast marching threshold-based level set. However, the low contrast between foreground and background made it difficult to stop the level set evolution. Additionally, the number of seed point could be normally up to 10–15 points, specified on 4–5 slices, to sufficiently capture their variations.

In^26^, a seed point was manually placed on IVC for extracting abdominal blood-vessels (ABV), which was classified into hepatic (HPV) and non-hepatic (non-HPV) blood-vessels. These vessels were then exploited for liver segmentation. This method achieved the highest score in its class. However, should any errors arise, interventions were required from the user. These included re-selecting the seed point, separating kidneys from a liver, untangling HBV from non-HBV, or removing IVC at the entry and exit points. Region growing was also preferred by methods in this group. Lu et al.^4^ adopted Quasi-Monte Carlo (QMC) method in selecting a seed point. Since low level features were considered, non-linear filter and morphological operation were needed for noise and over-segment removal, respectively. Following our literature survey, graph-cut was also found prevalent. Peng et al.^27^ proposed an appearance model-based approach, in which a liver was divided into multiple sub-regions. To begin with, initial regions were manually specified in a cylinder shape. Graph-cut, whose optimization was based on a geodesic distance, was then used to extract a liver surface. Similar work was proposed by Liao et al.^28^. Unlike^27^, the cost function was derived from intensity and appearance models. Bottleneck detection method was finally employed to remove any false positive. Without subject-specific training set, a deformable model could be built from manually drawn contours^29^ on some slices. In that study, a 3D liver was approximated by interpolating these contours. Each vertex on this model was matched to a feature point in the underlying image. For post-processing, a visual-based tool was provided for a user to finely adjust the segmentation results.

## Summary of latest liver segmentation algorithms

It is worth noted from the above rigorous investigations that, fully-automatic approach generally relied on statistically trained appearance models or on insights into liver morphology^10–16^. In the absence of such information, semi-automatic segmentation required a user as a domain expert, to provide initial seed points, contours, surfaces, etc., in training a classifier^18^, during subsequent processes^17,19–22^, to make final adjustment^4,23–29^. Due to inter-and intra-observer variabilities associated with these methods, extent to which user interaction was involved is one of the key determinants in benchmarking. A summary of state-of-the art methods is presented in Table 1a and b.

**Table 1:** Characteristics of early works on automatic methods (a) and semi-automatic methods (b).

Method	Techniques	Remarks	Dataset	Results
**(a) Automatic methods**				
Heimann 2007^10^	SSM	Trained by 20 instances	MICCAI	Score: 73
Kainmüller 2007^11^	SSM	Trained by 112 instances	MICCAI	Score: 59
Chen 2012^12^	AAM and LW	Training set required Validated by Leave-One-Out Strategy	MICCAI (Labelled) Private	VOE: 6.5% TPVF > 94% FPVF < 0.2%
Li 2015^13^	SSM and Deformable GC	Alternately trained and tested between two datasets	MICCAI 3D-IRCADb	VOE: 6.2% VOE: 9.15%
Lu 2017^14^	CNN and GC	Trained by 78 instances	MICCAI (Unlabeled) 3D-IRCADb	Score: 77.8 VOE: 9.36%
Hu 2016^15^	Deep Learning	Trained by 109 instances	MICCAI (Unlabeled) Private	Score: 80.3 DICE: 97.2%
Li 2018^16^	2D DensetUNet and 3D DenseUNet	Trained by 131 instances High computational demand	3D-IRCADb Liver and Tumor LiST	DICE: 98.2% DICE: 93.7% DICE global Liver: 96.5% DICE global Lesion: 82.4
Selver 2008^17^	K-Means and MLP	Significant user intervention. Required non-linear filter and morphology operator to refine the resultant segments	Private	Success rate: 94.9%
Zheng 2017^18^	GLCM and Random Walks	Trained by 18 2D instances Time consuming operations	MICCAI	Score: 76
Marcin 2014^19^	Contour Approximation	Dependent on lumbar spine location and the symmetry of input image. Highly sensitive to noise	Private (2D)	DICE: 81.3%
Wu 2016^20^	MIP, Super Voxel and GC	Contour initialization where large tumors present may not be reliable	MICCAI (Labelled) MICCAI (Unlabeled)	Score: 752 Score 71.4
Kumar 2011^21^	Region growing and Fuzzy C-Mean	Seed point localization may be inaccurate in noisy image	Private	Liver: spatial overlap: 0.98 Lesion: spatial overlap: 0.94
Huang 2018^22^	K-Means and GC	Sensitive to size and number of tumors. Hole filling needed	MICCAI (Unlabeled) 3D-IRCADb	VOE: 5.3% VOE: 8.6%
**(b) Semi-automatic methods**				
Dawant 2007^23^	Level set	Moderate user interaction	MICCAI	Score: 76
Lee 2007^24^	Level set	Minimal user interaction	MICCAI (Unlabeled)	Score: 75
Yang 2014^25^	Level set	Initialization required 2 to 15 point on each of 4 to 5 slices	MICCAI (Unlabeled) Private	Score: 78.9 SI = 97.6%
Maklad 2013^26^	Region growing and ABV	User interaction required	MICCAI (Unlabeled)	Score: 85.7
Lu 2014^24^	Enhanced region growing and QMC	Pre-processing and morphological operations were required for noise removal and over-segments correction, respectively	Private (2D)	Visual Assessment
Peng 2015^27^	GC with Geodesic distance cost	Multiple seed points were manually initialized within a cylindrical shape	MICCAI (Labelled) MICCAI (Unlabeled) Private	Score: 83.3 Score: 83.4 DSC: 97.5%
Liao 2016^28^	GC with Shape constraints	GC energy terms were built from a patch of liver. The shape constrains was imposed within a same slice	MICCAI: (Labelled) Private	VOE: 5.8% DSC: 97.3% VOE: 5.5% DSC: 97.2%
Chartand 2017^29^	Deformation model by Laplacian mesh optimization	User interaction was required to refine segmentation results	MICCAI Private	VOE: 5.1% VOE: 7.6%

## Proposed method

**Figure 2:**
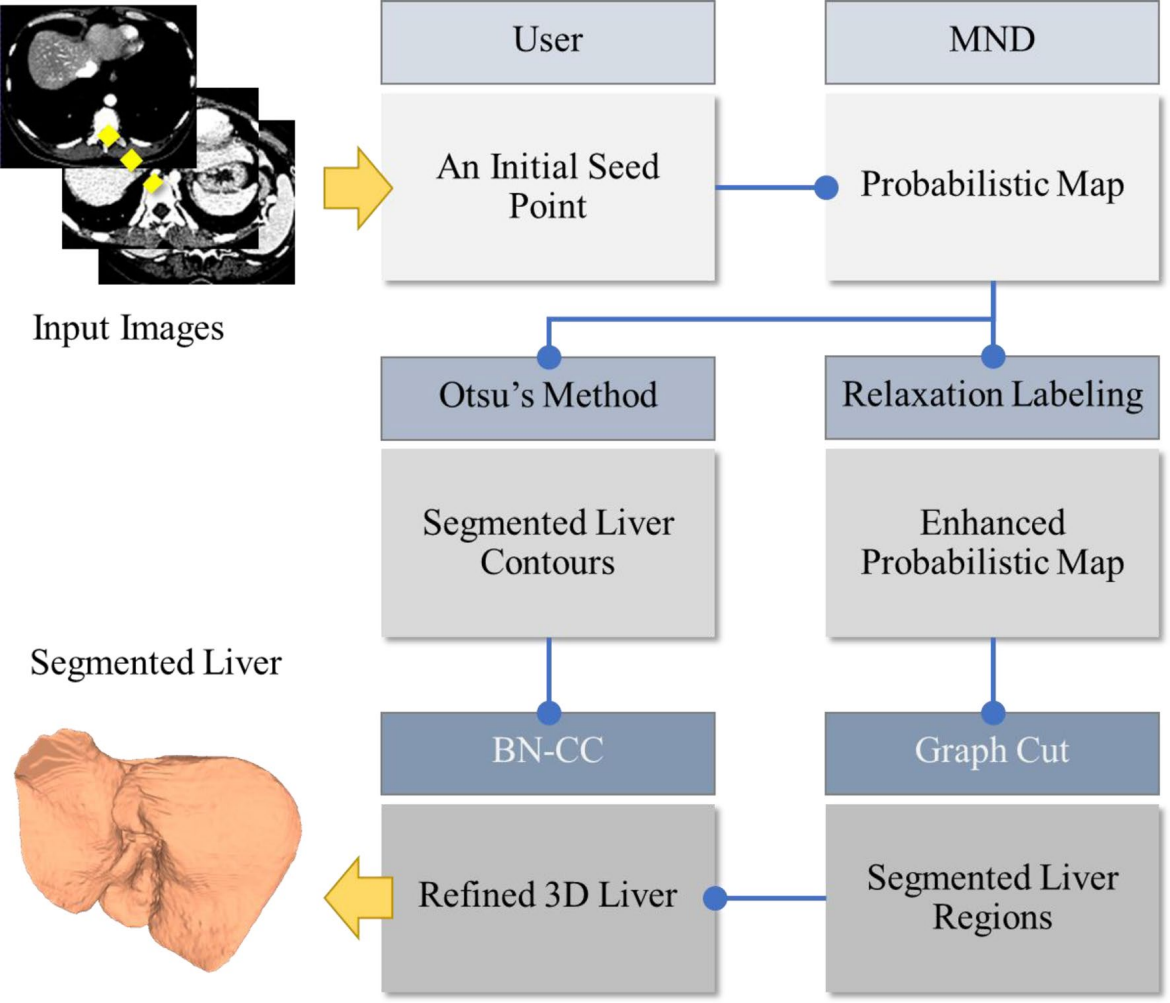
Diagram of the proposed scheme. Herein, MND and BN-CC stands for multivariate normal distribution and bottleneck detection and contour constraint, respectively.

Probabilistic models have been investigated in many image analysis studies and demonstrated reliable performance. Balance between model richness and simplicity was however of primary concern in practical settings. Since CT pixels are strictly calibrated in Hounsfield Unit (HU), this paper could assume trivial normal distribution of intensities and considered up to 2nd order statistics in building pixel-wise probabilistic map. Subsequently, dual image segmentations were run in parallel. Firstly, relaxation labeling (RL) took into account spatial context and iteratively enhanced the map, i.e., removing outliers while aggregating dispersed objects. Subsequently, this fully connected network was fed into GC process to optimize liver versus background separation. In another parallel process, Otsu’s method was applied to the pre-optimized map to delineate a set of liver contours. Both region and boundary segments were finally coupled and imposed with heuristic anatomical constraints by bottleneck detection and adjacent contours similarity. The proposed scheme was summarized in Fig. 2. Each involving process is described in the subsequent sections.

The proposed model was constructed by a distribution of random variables whose members are statistical properties within a local neighborhood of each pixel (instead of its intensity) in a small patch, surrounding user defined seed point. Compared to similar studies^14,15,18^, it was simpler to evaluate but similarly robust against imaging noise. Thus, pre-processing for noise removal was not necessary^4,17,19,21,24^. Furthermore, it neither required priors on liver appearance nor its morphology. For post-processing, contextual and anatomical constraints were imposed, respectively, by RL and BN-CC, instead of arbitrary morphological operator^13,17,20,24^ and manual adjustment^17,29^.

### Multivariable normal distribution model

Multivariable normal distribution (MND) of image features has been widely adopted in varius computer vision problems. Its applications include linear colour transformation^30,31^, classification^32^, and restoration^33^. In this paper, the model was used to build a probability map of liver tissues for preliminary classification. A probability of a point $\mathbf{p}$ being of liver was determined based on a patch ($\Phi$) of size $m\times n$ centered by that point. To begin with, for each pixel ${\mathbf{q}}_{i}\in\Phi$, the local mean (${\mu }_{i}$) and standard deviation (${\sigma }_{i}$) of the intensities, within its $m\times n$ neighbors ($\Omega$) are computed. In this study, the extent of neighbors in x and y directions, i.e., *m* and *n*, respectively, were equally set to 11 pixels (approx. 2.75–4.00 mm, either side). The local (${\mu }_{i}$) and standard deviation (${\sigma }_{i}$) were then averaged over the members, ${\mathbf{q}}_{i}\in\Phi$. The resultant averages constituted to a 2D vector values characterizing the given point $\mathbf{p}$, as expressed in Eq. (1).

Let a vector function **f**: **R**^2^ → **R**^2^ map a point $\mathbf{p}$ to its feature space as follows:

1 $$\mathbf{f}\left(\mathbf{p}\right)={\left[\begin{array}{cc}\frac{\sum_{i\in\Phi }{\mu }_{i}}{\Vert \Phi \Vert }& \frac{\sum_{i\in\Phi }{\sigma }_{i}}{\Vert \Phi \Vert }\end{array}\right]}^{T}$$

where local mean (${\mu }_{i}$) and standard deviation (${\sigma }_{i}$) were evaluated for each ${\mathbf{q}}_{i}\in\Phi$ over its neighbors ($\Omega$).

A normal distribution of a *k*-dimensional random variable (RV), expressed by $\mathrm{f}={[{f}_{1},{f}_{2},\dots ,{f}_{k}]}^{T}$, was defined as:

2 $$\mathcal{P}\left(\mathbf{f}\right)=\frac{1}{{2\pi }^{k/2}{\left|\Sigma \right|}^{1/2}}{e}^{-\frac{1}{2}{\left(\mathrm{f}-\stackrel{-}{\mathrm{f}}\right)}^{T}{\Sigma }^{-1}(\mathrm{f}-\stackrel{-}{\mathrm{f}})}$$

where $\stackrel{-}{\mathbf{f}}\in {\mathrm{R}}^{k},\Sigma \in {\mathrm{R}}^{k\times k}$ were the mean and covariance matrices of **f**, respectively. It was computed from a set of few manually specified points. In this study, they were evaluated by (1) at the seed point. Provided the definition of a vector function of feature, **f** (**p**) = [*f*_1_ *f*_2_]^*T*^, having *k* = 2 dimensions, the corresponding covariance matrix was thus given by:

3 $$\Sigma =\left[\begin{array}{cc}cov\left({f}_{1},{f}_{1}\right)& cov\left({f}_{1},{f}_{2}\right)\\ cov\left({f}_{2},{f}_{1}\right)& cov\left({f}_{2},{f}_{2}\right)\end{array}\right]$$

The process of building a probability density function (pdf) on a given image *I* is described in Table 2.

**Table 2:**
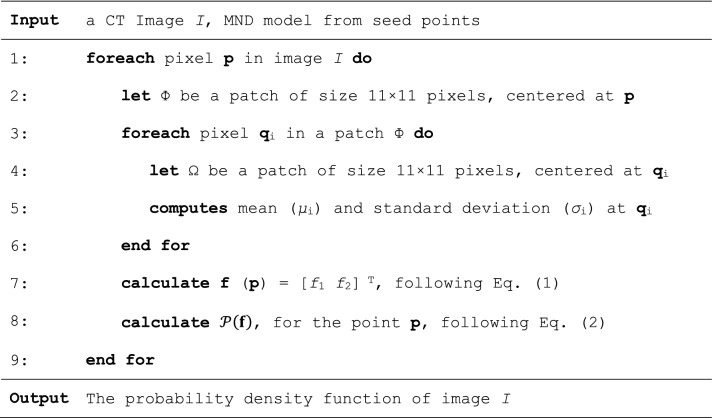
Process of building a multivariate probability density function on an image.

To highlight minimal user intervention, the following experiments was made on an MND built from single patch, surrounding a user defined point, **p**. It is also worth raised here that, if pixel intensities (instead of their local statistics) were considered as the random variable, the resultant MND would have been much sensitive to inherent imaging noise. Accordingly, it would require more or larger patches to capture variations due to inhomogeneity, or else pre-processing for noise removal^4,17,19,21,24^. In addition, for a typical liver study, display range is normally set to W:150 and L:80. This setting leads to low contrast among abdominal organs. Thus, building MND from pixel intensities would be dependent on viewing parameters, otherwise a full range of HU values would be needed, in which case, noise in those range would be inevitably included.

### Relaxation labeling

Relaxation labeling (RL) is a simple tool that can robustly solve a multi-class labeling problem. Given an initial probabilistic map of MND, the RL iteratively adjusts the labeling of an object, based on contextual information, inferred by its neighbors^34^. The contexts include supports from within and inter-class memberships of other objects within a given proximity. To encourage robustness (or rapid convergene), a damping (or accelerating) factor can be incoproated into the updating formula. With these advantages, RL has been exploited in many applications, e.g., image segmentation^35,36^ line and curve enhancement and point matching^37^. Unlike other works, RL was adopted here to improve initial pixel-wise classification, obtained from the prior step. Since basic elements and their definitions can be found in^34^, this section elaborates in detail only supports and compatibility functions.

Herein, objects having their probability updated were pixels in a given CT image, **I**. Let the probability of a pixel **p** ∈ **I** belonging to a class $\lambda \in \mathcal{C}$ at an iteration *t* be desfined as ${\mathcal{P}}_{p}^{t}(\lambda )$. Then, the updating of the probability at the next iteration, *t* + 1, is calculated by Eq. (4) ^30^.

4 $${\mathcal{P}}_{\mathbf{p}}^{t+1}(\lambda )=\frac{{\mathcal{P}}_{\mathbf{p}}^{t}(\lambda )\left(1+{S}_{\mathbf{p}}\left(\lambda \right)\right)}{\sum_{\mu \in \mathcal{C}}{\mathcal{P}}_{\mathbf{p}}^{t}(\mu )\left(1+{S}_{\mathbf{p}}\left(\mu \right)\right)}$$

where ${S}_{\mathbf{p}}\left(l\right)$ is the support function for pixel $\mathbf{p}$ by a label, *l*.

Let ${\mathcal{N}}_{\mathbf{p}}$ be a set of neighbors of $\mathbf{p}$ and ${r}_{\mathbf{p}\mathbf{q}}(\lambda ,\mu )$ be the compability between pixels $\mathbf{p} \mathrm{and} \mathbf{q}\in {\mathcal{N}}_{\mathbf{p}}$ by labels $\lambda$ and $\mu$, respectively. The support function was derived from compatibility, given by Eq. (5).

5 $${S}_{{\varvec{p}}}\left(\lambda \right)=\sum_{\mathbf{q}\in {\mathcal{N}}_{\mathbf{p}}}{w}_{\mathbf{p}\mathbf{q}}\sum_{\mu \in \mathcal{C}}{r}_{\mathbf{p}\mathbf{q}}\left(\lambda ,\mu \right){\mathcal{P}}_{\mathbf{q}}^{t}\left(\mu \right)$$

where ${r}_{pq}\left(\lambda ,\mu \right)$ was 1, if λ and µ were of the same class, or 0, otherwise. The inter-object weight ${w}_{\mathbf{p}\mathbf{q}}$ was defined as an inversed Euclident distance between **p** and **q**. It was also normalized such that its sum over the neighbours ${\mathcal{N}}_{\mathbf{p}}$ were unity.

**Figure 3:**
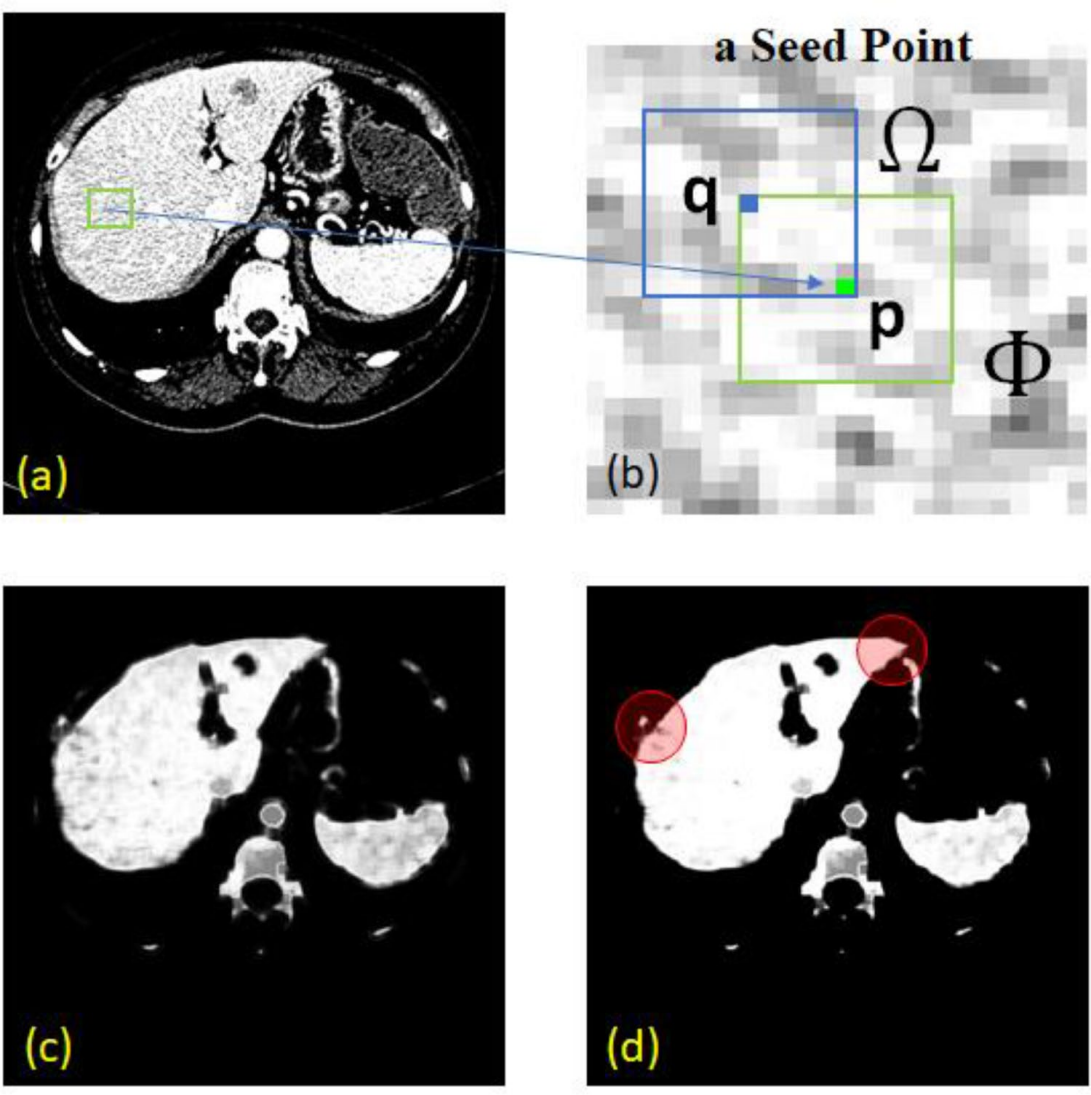
An example of applying probability map and relaxation labeling. The original CT image (**a**) with a seed point (green) is shown in (**b**), the probability map image and RL enhanced results are shown in (**c**) and (**d**), respectively. The probability values from 0 to 1 are scaled to 0 to 255 for visualization purpose.

To ensure the performance of the system, the neigbour size was set to only within one pixel proximity in 8 directions. It is also worth noted that, with trivial features, pixel-wise classification may result in vague definitions along connective tissues. To sufficiently enhance such separation prior to the next stage, probabilistic convergence and hence finalized labeling, was not yet required here. The RL was therefore allowed to update, not until convergence, but only for a few iterations. Figure 3 depicts an example configuration of pixel **p** and its neighbour **q**, the initial probabilistic map, and the resultant RL enhancement. It is clear that fallacies along the boundary were effectively reduced (red circles).

### Graph cut

The graph-cut algorithm has recently attracted interests in various systems for medical image segmentation. It was posed as a graph optimization problem, whose cost functions are defined both on regions and boundaries of object^13,14,20,27,28^. Particularly for solving binary segmentation, a max-flow/ min-cut algorithm, proposed in^38^, was proved efficient, and thus employed in this work.

Consider a set of pixels in an image $I$, each pixel $\mathbf{p}\in I$ is assigned with a binary label ${\mathcal{C}}_{\mathbf{p}}\in \left\{0, 1\right\}$, whether it belongs to background or an object of interest, respectively. Segmenting the object is then defined as determining a set of individual labels $\mathcal{L}=[{\mathcal{C}}_{1}, {\mathcal{C}}_{2},\dots ,{\mathcal{C}}_{\left|I\right|}]$, assigned to each pixel, such that it minimizes an energy function,

6 $$E\left(\mathcal{L}\right)=\alpha \sum_{\mathbf{p}\in I}R({\mathcal{C}}_{p})+\left(1-\alpha \right)\sum_{\mathbf{p}\in I, \mathbf{q}\in {\mathcal{N}}_{p}}B\left({\mathcal{C}}_{p},{\mathcal{C}}_{q}\right)$$

where $\alpha$ was a balancing weight between the region and boundary terms. In the following experiment, it was set to 0.50. The size of labels, |* I* |, equaled the number of pixels in the image. ${\mathcal{N}}_{p}$ is defined following that in previous section. The log conditional probabilities in Eq. (6) was given as follow:

7 $$R\left({\mathcal{C}}_{\mathbf{p}}=c\right)=-\mathrm{ln}\mathcal{P}\left(\mathbf{p}|c\right)$$

where *c* was a binary label assigning 0 or 1 to either a background or object pixel, respectively. The probabilistic map $\mathcal{P}\left(p\right)$ was obtained from the previous stage (Eq. 4). The boundary term was directly calculated from the intensities of adjacent pixels and the Euclidean distance between them, as follows:

8 $$B\left({\mathcal{C}}_{p},{\mathcal{C}}_{q}\right)=\mathrm{exp}\left(-\frac{{\left({I}_{\mathbf{p}}-{I}_{\mathbf{q}}\right)}^{2}}{2{\sigma }^{2}}\right)\frac{1}{dist(\mathbf{p},\mathbf{q})}$$

where $\sigma$ was the noise distribution, estimated from the CT image.

### Post-processing

**Figure 4:**
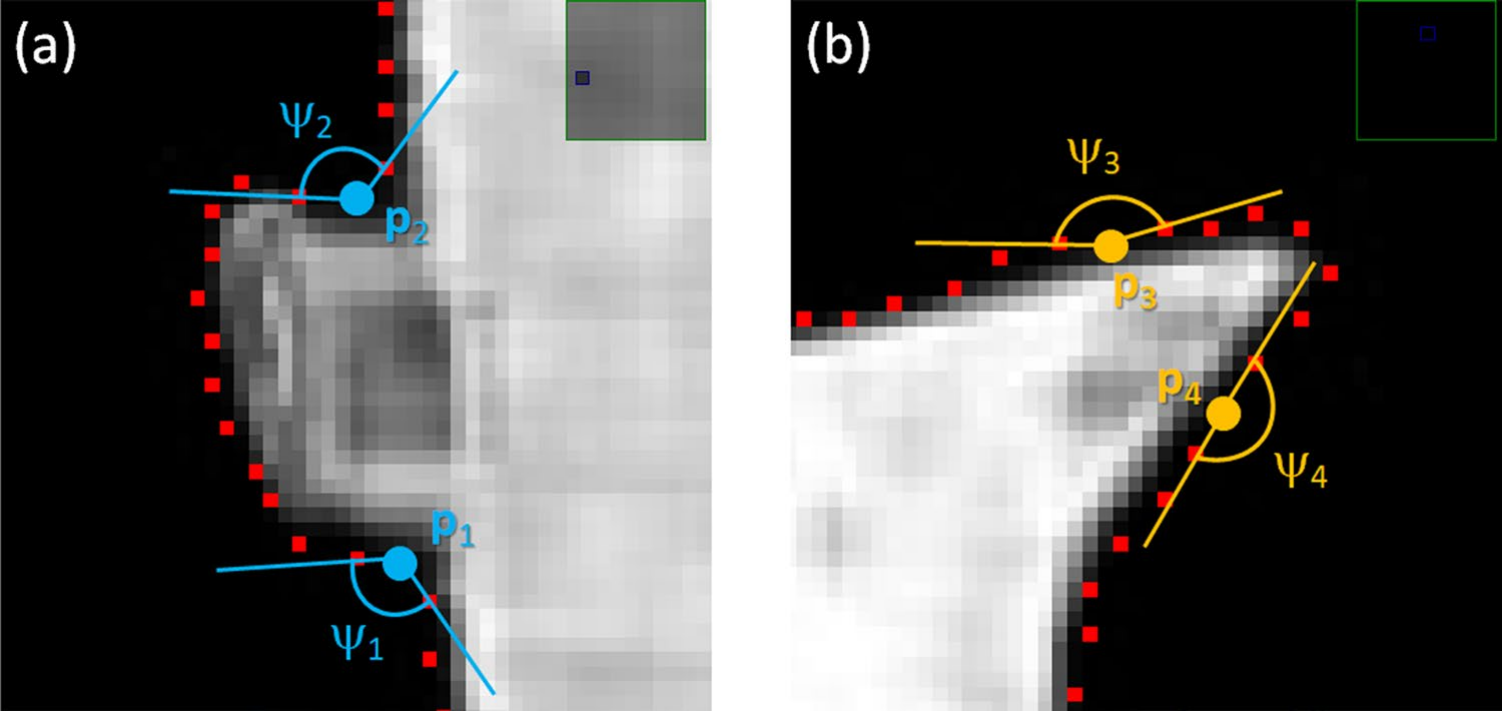
Some examples of bottleneck detection. The exterior angles of the potential points ${p}_{1}, {p}_{2}, {p}_{3}, {p}_{4}$ are 110°, 120°, 161°, and 180°, respectively. The cost function in both (**a**) and (**b**), computed by Eq. (9) were $\mathcal{E}\left({p}_{1},{p}_{2}\right)=0.5$ and $\mathcal{E}\left({p}_{3},{p}_{4}\right)=0.4$, respectively.

RL enhanced probabilistic map and graph-cut provided a reliable and efficient segmentation of the liver, based on intensity distribution of the pixels and their spatial relationship. Thus far, due to rather complex geometry of the liver and its similar X-ray absorption properties to other organs, there remained over-segmentation. This led to low accuracy, commonly found in many existing untrained systems or those trained with inadequate samples. It was observed that over-segments often appeared as nodal shapes on the liver boundary. To further improve the results, instead of manual editing, this paper thus imposed an anatomical control over the segmented result, based on bottleneck detection and contour constraint (BN-CC). According to Wang et al.^39^, a potential bottleneck in 2-dimensions space is determined by a cost function $(\mathcal{E})$, defined by a pair of points (**q**, **p**) and **p** ≠ **q**, such that,

9 $$\mathcal{E}\left(\mathbf{p},\mathbf{q}\right)=\frac{dist(\mathbf{p},\mathbf{q})}{\mathrm{min}(aL\left(\mathbf{p},\mathbf{q}\right), aL\left(\mathbf{p},\mathbf{q}\right))}<{\mathcal{T}}_{b}$$

where *dist* (**p**, **q**) was Euclidean, while the lengths aL (**p**, **q**) and aL (**q**, **p**) were the arc-lengths in clockwise direction along the contour. ${\mathcal{T}}_{b}$ is a predefined threshold. For a liver shapes, it was typically set to 0.60. A drawback of the method was that as the threshold increased, it tended to smooth out the contour. In some instances, anatomical features such as that on the left lobe was partially brushed off. On the other hand, reducing the value caused substantial over-segments, mostly near the ligaments. In addition, to avoid these concerns on contour modeling, a generic polygonal approximation was applied to extract key points. Depending on image resolution, this may result in too many points being generated. To simultaneously tackle these problems, this study introduced a criterion on a candidate point based on its exterior angle. Specifically, if only its exterior angle was less than a given threshold, it would be considered in bottleneck detection, otherwise it remained on the segmented contour. In this study the threshold for nodal point candidate was set to 150°. Figure 4 illustrates two examples of candidate point selections. Segments (**p**_1_, **p**_2_) and (**p**_3_, **p**_3_) had cost functions of 0.50 and 0.40, respectively, and would be both identified as bottlenecks. As such, this would have incorrectly removed a salient point at the end of left lobe (b). However, only the segment (a) (fuzzy liver edge) satisfied the nodal candidate exterior angle criterion and hence was removed but leaving the segment (b) untouched.

**Figure 5:**
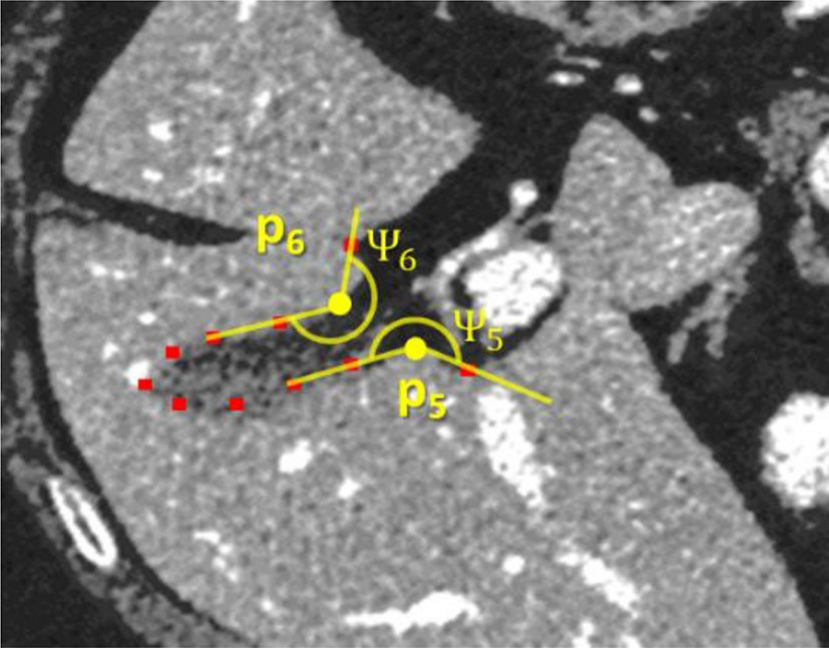
Both vertices have cost function of 0.25, which would be considered as BN by Eq. (9), but their exterior angles are both larger 150° threshold. Therefore, they are correctly excluded from BN candidates.

Unlike BN rate employed in^28^, the proposed exterior angle was beneficial in constraining the correct direction of nodal segments. Figure 5 illustrates two vertices, **p**_5_ and **p**_6_, whose $\mathcal{E}\boldsymbol{ }\left({{\varvec{p}}}_{5},{{\varvec{p}}}_{6}\right)=0.25$, but their exterior angles are 210° and 245°. They would then correctly be excluded from BN candidates.

Nonetheless, it was found during our preliminary experiment that depending on the thresholds, not all bottlenecks could be successfully removed. Information on adjacent slices was thus also considered in post-processing. With modern CT imaging, slice thickness was typically small, and object shapes do not differ much between adjacent planes. Thus, a contour constraint was imposed on the segmented regions. To begin with, each probabilistic map of two adjacent slices were first converted to binary images, by using an Otsu’s method. Complementary contours would be extracted from these images. Let ${C}_{i}=\left\{{c}_{i1},{c}_{i2},\dots \right\}$ and ${C}_{j}=\left\{{c}_{j1},{c}_{j2},\dots \right\}$ be the sets of contours (including all bottleneck candidates), extracted from *i*th and *j*th slices, respectively. For any $\left({c}_{ik}, {c}_{jl}\right)\in {C}_{i}\times {C}_{j}$ that satisfied the condition,

10 $$\mathcal{S}=\frac{\left|{c}_{ik}\cap {c}_{jl}\right|}{\mathrm{min}\left(\left|{c}_{ik}\right|,\left|{c}_{jl}\right|\right)}<{\mathcal{T}}_{c}$$

then the contour with the least area, i.e., | *c*_*mn*_ |, would be removed from the respective slice. In our preliminary experiment, a suitable threshold was empirically estimated by using simple linear least-square method. The suggested threshold ${\mathcal{T}}_{c}$ was given as a function of slice distance, dz.

11 $${\mathcal{T}}_{c}=0.8-0.05({d}_{Z}-1)$$

**Figure 6:**
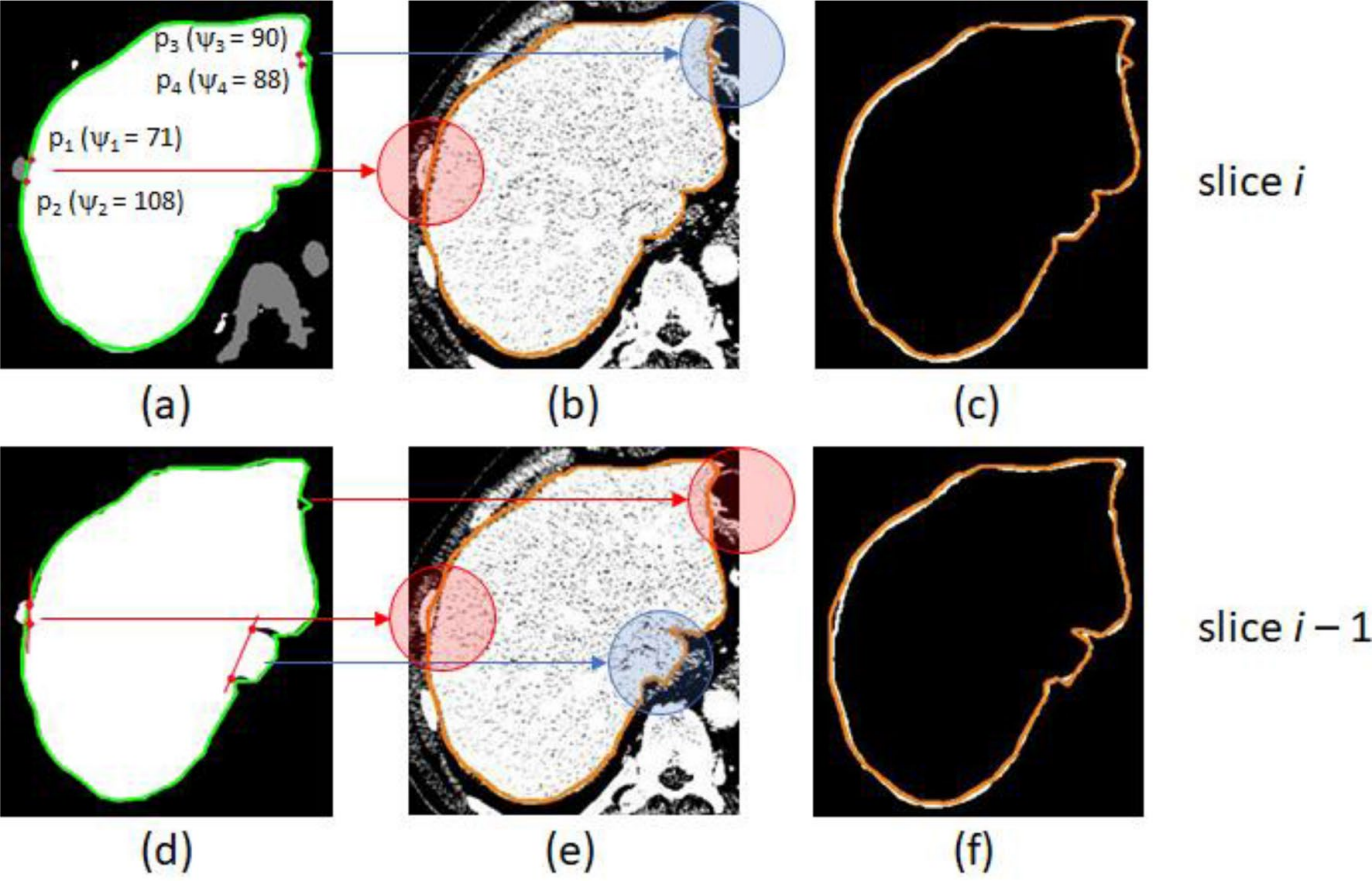
The performance of BN-CC shown on two consecutive slices (top and bottom rows). The previous and current contours are draw in green and orange colors, respectively. The ground truth is drawn in white. The first column (**a** and **d**) shows the bottle neck detecting in CT image after applying graph-cut. The second column (**b** and **e**) shows the results after applying adjacent contour constraint. The last column (**c** and **f**) shows the valid contours. Red and blue circles indicate the removed and remaining bottlenecks, respectively.

To avoid inconsistency due to slice orders, post-processing started from a slice with the largest contour, and stepped one slice at a time in both directions along the z axis. For any pair of slices being processed, BN-CC was first applied, following Eq. (9) and remaining contours were constrained, i.e., removed subject to the condition, given by Eq. (10) and (11). Figure 6. demonstrates some examples of approximated contours from slices *i* and *i* − 1 and those after applying BN-CC. In the top row (a)–(c), there were 2 bottlenecks (**p**_1_, **p**_2_) and (**p**_3_, **p**_4_), whose areas were 242 and 52 pixels, respectively. They would be both detected by Eq. (9). When intersecting with the one in previous slice, whose area was 42,670 pixels, the intersected areas were 42 and 51 pixels, respectively. With ${\mathcal{T}}_{c}$ set to 0.8 (i.e., d_z_ = 1), only the former would be removed (circled in red), leaving the latter (circled in blue). Likewise, not all bottlenecks were removed by Eq. (9) in the bottom row (d–(e), unless they satisfied Eq. (10).

### Ethical approval

This article does not contain any studies with human participants or animals performed by any of the authors.

## Experiments

The proposed technique was developed by using C and C +  + languages and implemented on Linux operating system. It ran on a personal computer equiped with a 2.4 GHz CPU and an 8 GB RAM. Basic image processing and graphics algorithms involved were derived from OpenCV^40^ and Visualization Toolkit (VTK) ^41^. Its performane is demonstrated by applying it on a public dataset, obtained from *MICCAI 2007 Grand Challenge* reprository. The dataset consisted of 30 CT volumes. Out of these, 20 volumes were training scans, whose ground-truth (labelled reference) was provided. The remaining 10 volumes, referred here as testing scans, were unlabeled. To evaluate the results on the latter, the authors were required to submit segmented livers to MICCAI SLIVER07 website. All images were recorded at a resolution of 512 × 512 pixels. The pixel sizes ranged from 0.55 mm to 0.8 mm, while distances between slices ranged from 1.0 mm to 3.0 mm. The number of slices in each volumetric scan varied between 64 to 502^3^. In order to visually assess the segmentation results, a surface model of segmted liver was reconstructed by using the Marching Cubes (MC), implemented in VTK^41^. The surface was rendered with false overlaid colors to represent error matrics. For quantitative evaluations, segmented liver volumes were compared against corresponding references, based on 5 evaluation metrics^3^. They were Volumetric Overlap Error (VOE), Relative Volume Difference (RVD), Average Symmetric Surface Distance (ASD), Root Mean Square Symmetric Surface Distance (RMSD), and Maximum Symmetric Surface Distance (MSD). The score for each metric was computed based on error rate $(e)$ and average user error ($\stackrel{-}{e})$, whose references were provided by^3^, over all instances. The higher these scores, the better the performance. Detailed descriptions of these metrices and the score are listed in Table 3.

**Table 3:** The definitions of five evaluation metrics.

Definitions	Units
$VOE=100\left(1-\frac{|A\cap B|}{|A\cup B|}\right)$	%
$RVD=100\left(1-\frac{\left|A\right|-|B|}{|B|}\right)$	%
$ASD=\frac{1}{\left|S\left(A\right)\right|+\left|S\left(B\right)\right|}\times \left(\sum_{{S}_{A}\in S(A)}d({S}_{A},S\left(B\right))+\sum_{{S}_{B}\in S(B)}d({S}_{B},S\left(A\right))\right)$	mm
$MSD=\sqrt{\frac{1}{\left|S\left(A\right)\right|+\left|S\left(B\right)\right|}}\times \sqrt{\left(\sum_{{S}_{A}\in S(A)}{d}^{2}({S}_{A},S\left(B\right))+\sum_{{S}_{B}\in S(B)}{d}^{2}({S}_{B},S\left(A\right))\right)}$	mm
$MaxD=max\left({max}_{{S}_{A}\in S\left(A\right)}d({S}_{A},S\left(B\right), {max}_{{S}_{B}\in S\left(B\right)}d({S}_{B},S\left(A\right)\right)$	mm
Score: $\Upsilon =max(100-25\frac{e}{\stackrel{-}{e}}$,0), with $\Upsilon \in [\mathrm{0,100}]$	

In this table, A, B, S (A), S (B) are the segmented volume, reference volume, the sets of voxels in the segmented and reference volumes, respectively. Out of the 20 CT scans, however, 18 ones were acquired from healthy subjects and those with minor lesions (referred to as **asymptomatic**). Since the proposed segmentation was specifically designed to work best on a normal liver, the images number 10 and 16, which contained extensive lesions were analyzed separately in quantitative assessments. Furthermore, to fully validate the proposed method, segmented livers from other 10 unlabeled scans were submitted to the SLIVER07 website. Out of these unlabeled scans, the returned metrics for 7 healthy and mildly conditioned cases were assessed. To maintain consistency throughout the experiments, window (W) and level (L) were fixed for all images, at typical liver display^42^, i.e., 150 and 80 HU, respectively.

## Results and discussion

Firstly, 18 asymptomatic livers from 20 labelled instances were segmented. Their VOE, RVD, ASD, RMSSD, MSD, and respective and overall scores are reported in Table 4 (except for the severe cases, i.e., 10 and 16). The average overall score for these instances is 72.3 ± 6.09. Six images (35%) had the scores higher than the average of the Grand Challenge submissions. Note the robustness against noisy data, as shown by a high score of 81.5 in image 05. Nonetheless, without appearance prior model, image 09 exhibited a relative low score, due to mostly obscure separation against other organs.

**Table 4:** The evaluation metrics obtained from 18 asymptomatic (labelled) cases.

CT Image	VOE		RVD		ASD		RMSSD		MSD		Overall score
	[%]	Score	[%]	Score	[mm]	Score	[mm]	Score	[mm]	Score	
01	10.9	*57.4*	0.9	95.1	1.9	*52.5*	3.6	*50.5*	30.6	60.4	63.2
02	7.9	69.3	1.3	93.0	1.3	66.7	3.0	57.9	32.9	57.3	68.8
03	6.7	73.8	− 0.4	97.6	1.0	75.8	2.2	69.9	19.2	75.1	**78.5**
04	6.8	73.6	− 0.7	96.3	0.9	76.8	1.5	**78.6**	14.4	**81.4**	**81.3**
05	6.3	75.6	− 0.4	97.9	0.8	79.5	1.6	77.8	17.8	76.9	**81.5**
06	8.2	68.0	0.3	98.5	1.3	67.9	2.9	60.2	24.6	68.1	72.5
07	8.9	65.1	− 1.1	94.2	1.6	61.2	2.9	59.2	23.4	69.7	69.9
08	9.4	63.4	2.8	85.0	1.7	56.8	3.1	56.7	29.2	62.2	64.8
09	9.6	62.3	− 3.3	82.5	1.7	56.6	3.5	51.0	29.7	61.5	*62.8*
11	7.3	71.6	0.1	**99.3**	1.4	65.8	3.1	56.4	24.1	68.8	72.4
12	6.8	73.4	1.7	91.1	1.0	74.5	2.0	72.6	23.4	69.6	**76.2**
13	10.5	58.8	6.9	63.5	1.6	60.9	2.4	66.9	18.2	76.5	65.3
14	7.4	70.9	2.6	86.0	1.2	69.1	3.2	54.9	31.9	58.6	67.9
15	5.6	**78.0**	2.2	88.0	0.8	**80.6**	1.6	77.1	17.3	77.6	**80.3**
17	7.8	69.6	2.0	89.6	1.2	68.9	2.8	60.8	23	70.2	71.8
18	8.2	68.1	2.9	84.8	1.3	68.4	2.1	71.5	20.2	73.8	73.3
19	6.4	74.9	1.8	90.7	1.1	72.2	2.7	63.1	38.2	*50.5*	70.3
20	6.2	75.7	1.1	93.9	0.9	78.2	1.7	75.9	17.5	77.3	**80.2**
Average	7.8	69.4	1.2	90.4	1.3	68.5	2.6	64.5	24.2	68.6	72.3
Std	1.5		2.1		0.3		0.7		6.6		6.09

The bold means the best values in each column.

**Figure 7:**
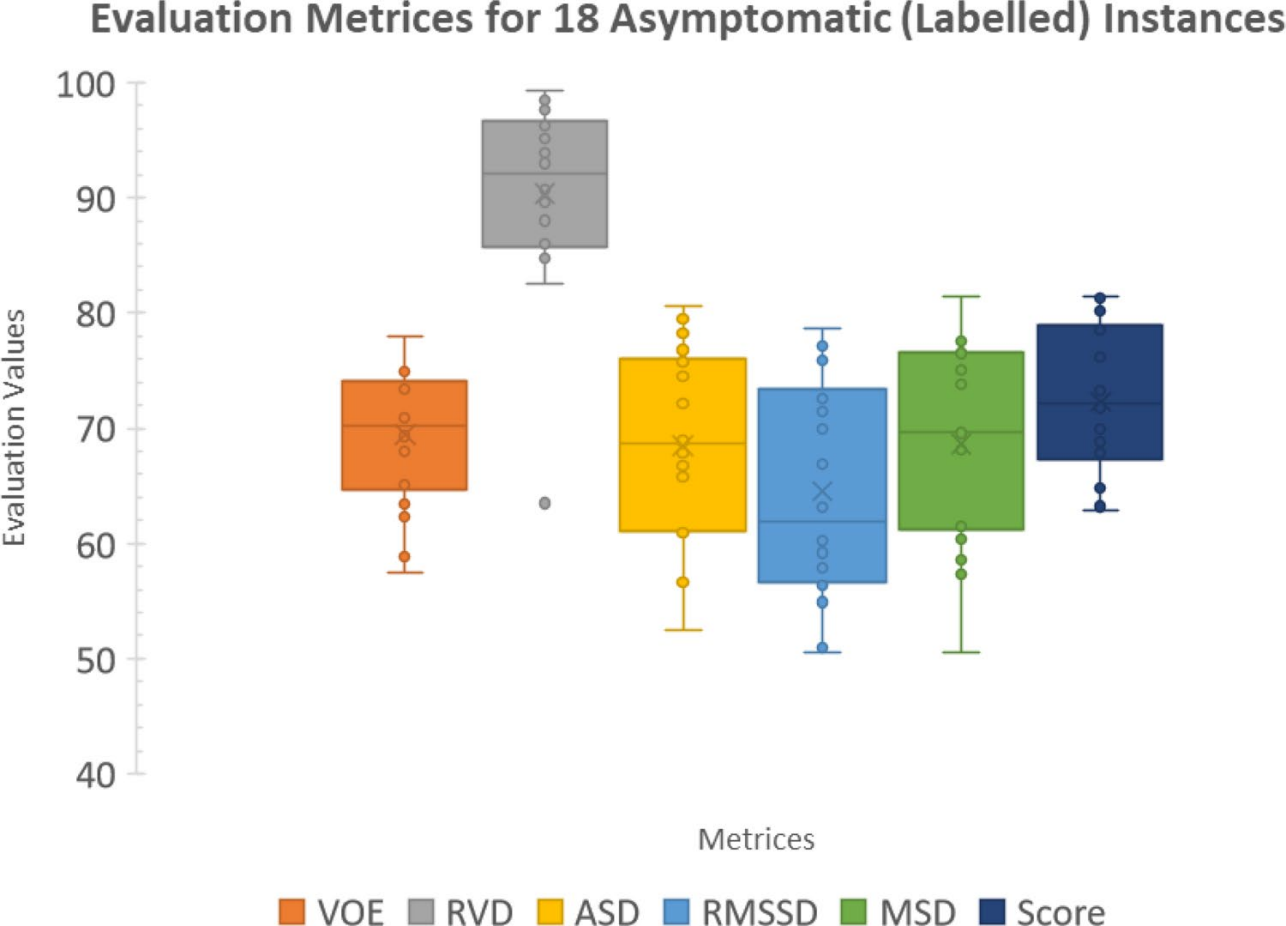
Box-Whiskers plots of VOE, RVD, ASD, RMSSD, MSD, and over scores for 18 asymptomatic images, drawn from labelled scans.

Figure 7 shows Box-Whiskers plots of these metrics and overall scores. Among these metrics, RVD was consistently the highest, followed by VOE and ASD, respectively.

**Figure 8:**
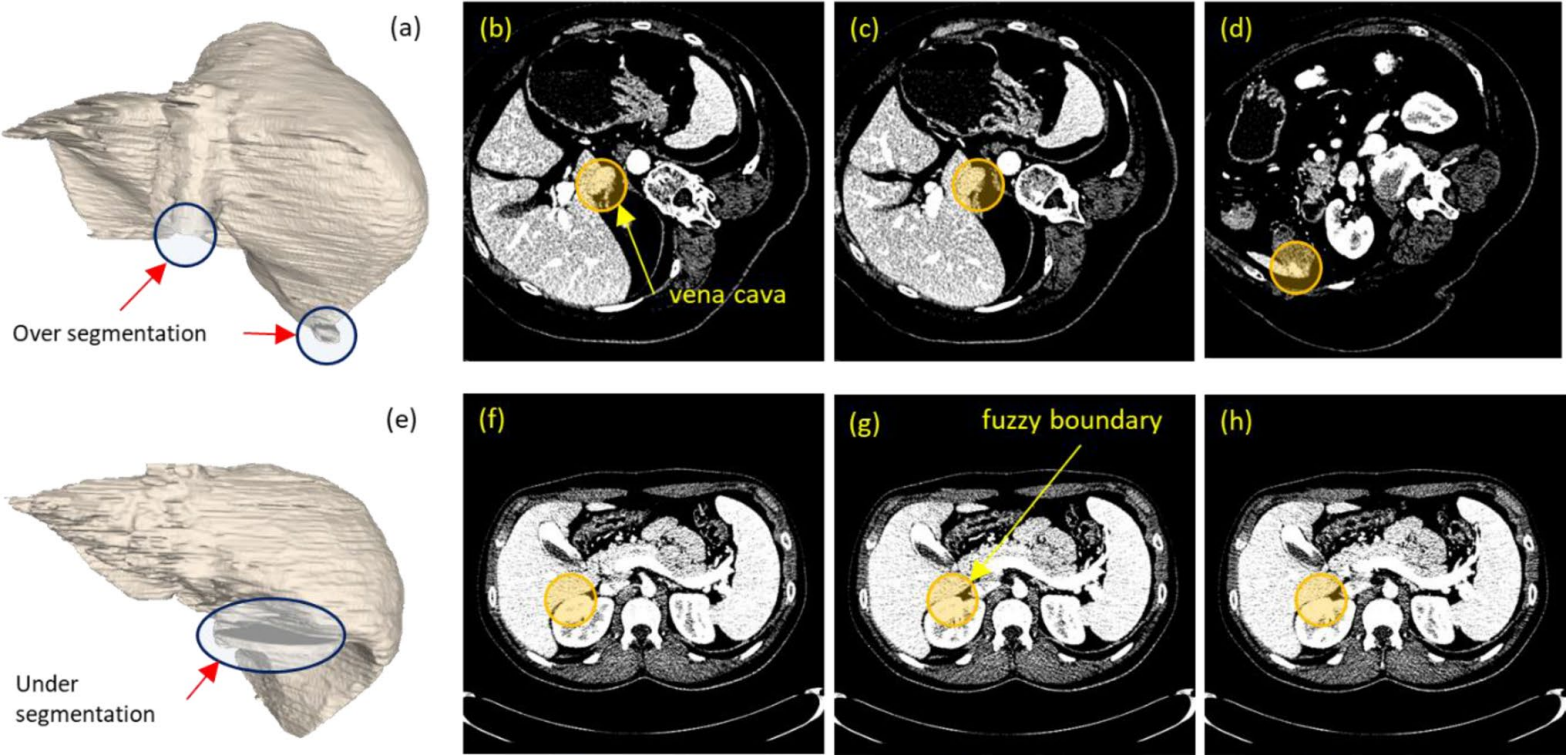
Examples of 3D segmentations (**a**) and (**b**), i.e., case 05 (top row) and 09 (bottom row), whose scores were the highest and the lowest, respectively. The corresponding images on their right shows CT slices where over (**b**), (**c**), and (**d**) and under (**f**), (**g**), and (**h**) segmentation occurred.

Figure 8 illustrates two cases, whose total scores were highest (05) and lowest (09), respectively, and their locations, where over and under segmentation occurred.

**Figure 9:**
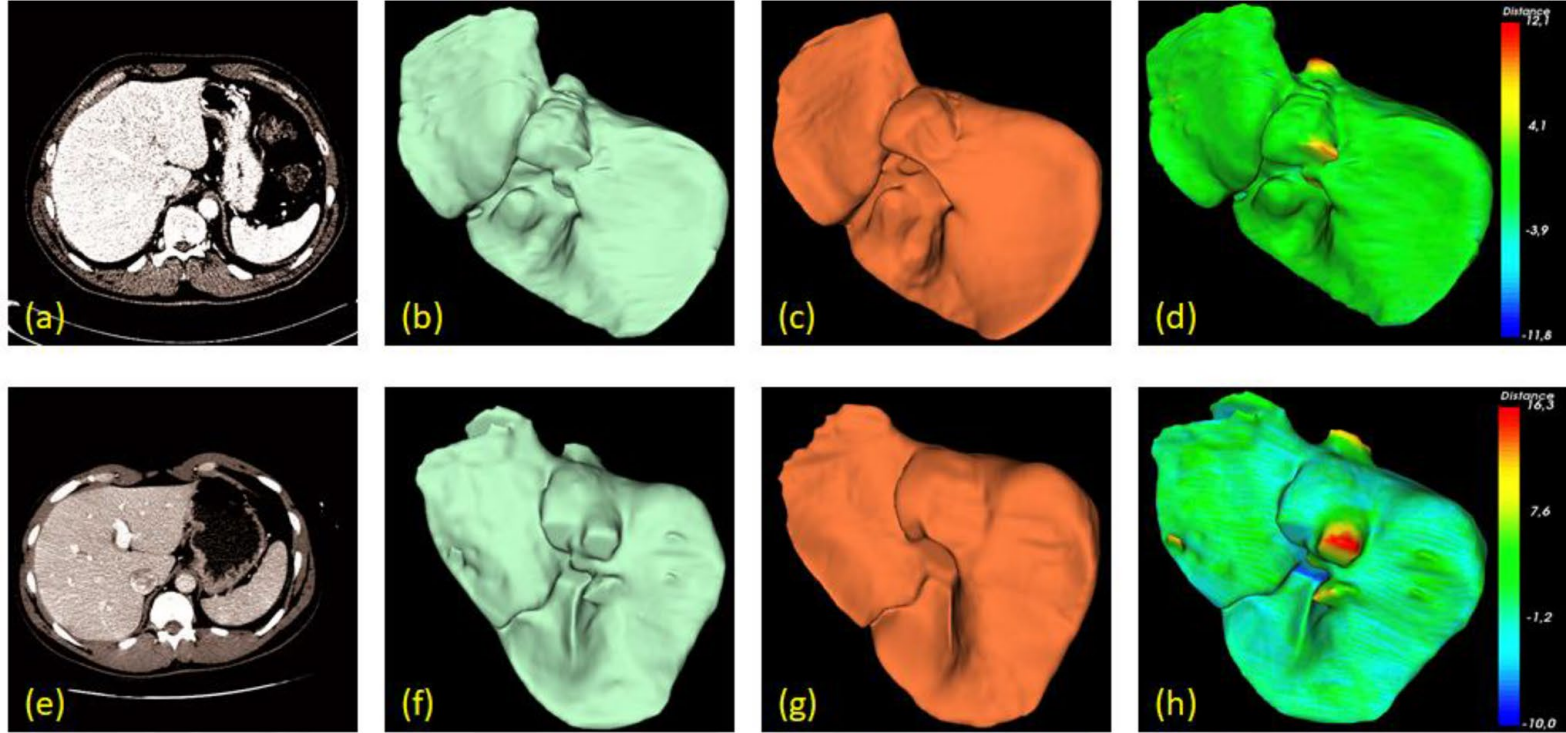
Selected examples of two healthy livers (top and bottom rows) in SLIVER07 dataset. The first column (**a**, **e**) shows an original image. The second (**b**, **f**) and third (**c**, **g**) columns show the segmented results and respective ground truths. The last column (**d**, **e**) shows the error distance (in mm) between our results and reference livers. The green color on the surface corresponds to the low error rate, while red and blue colors correspond to the high positive and negative ones, respectively.

Figure 9 illustrates 2 examples of segmented healthy livers (one row for each case) by the proposed method, compared with the respective ground truths. Each column depicts an original image, segmented liver, ground-truth, and respective surface, rendered with false colors, representing errors (in mm).

**Figure 10:**
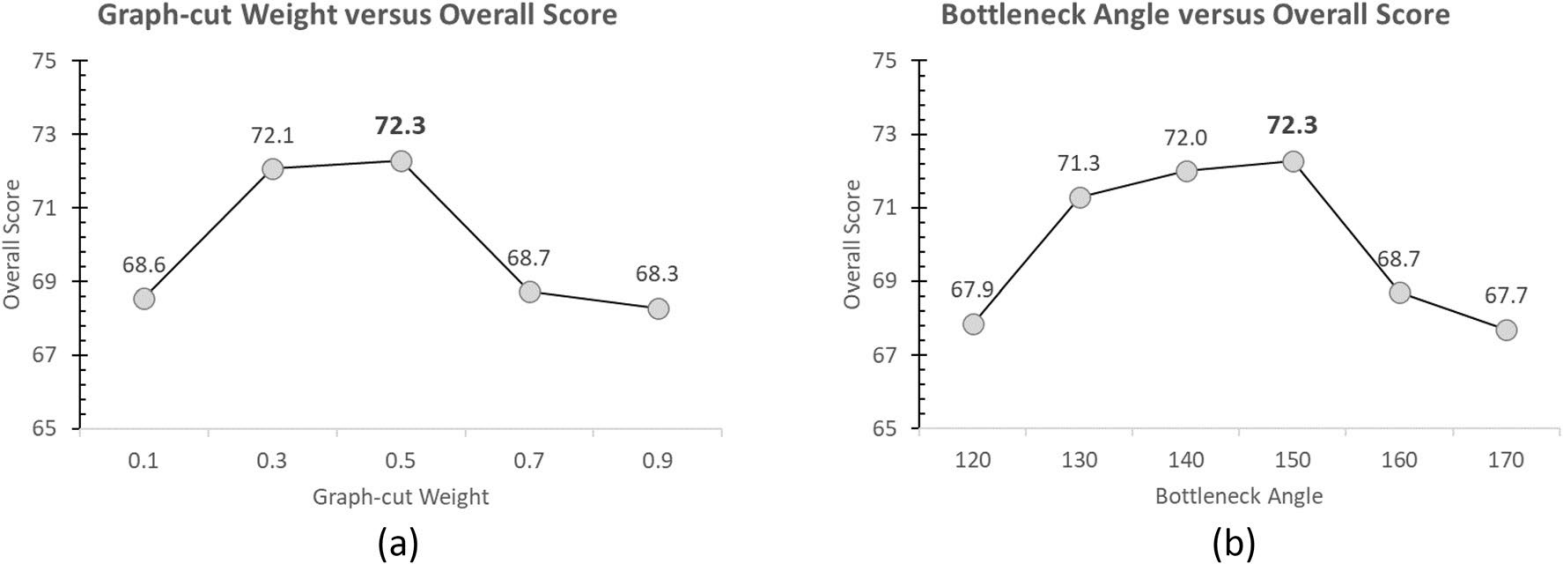
(**a**) Relationship between graph-cut weight and over score, showing the highest value at 0.5. (**b**) Relationship between bottleneck angle and over score, showing the highest value at 150.

Except a seed point, initialized by the user, the remaining process was fully automatic. However, there were two empirical parameters involved in the process, i.e., the weighting factor in graph-cut and the threshold angle for bottleneck condition. As a guideline on how to determine the appropriate values, the experiments were run on available dataset. The weight and threshold were varied between 0.1–0.9 and 120°–170°, respectively. Figure 10 plots the overall scores versus weights (a) and thresholds (b), respectively.

**Figure 11:**
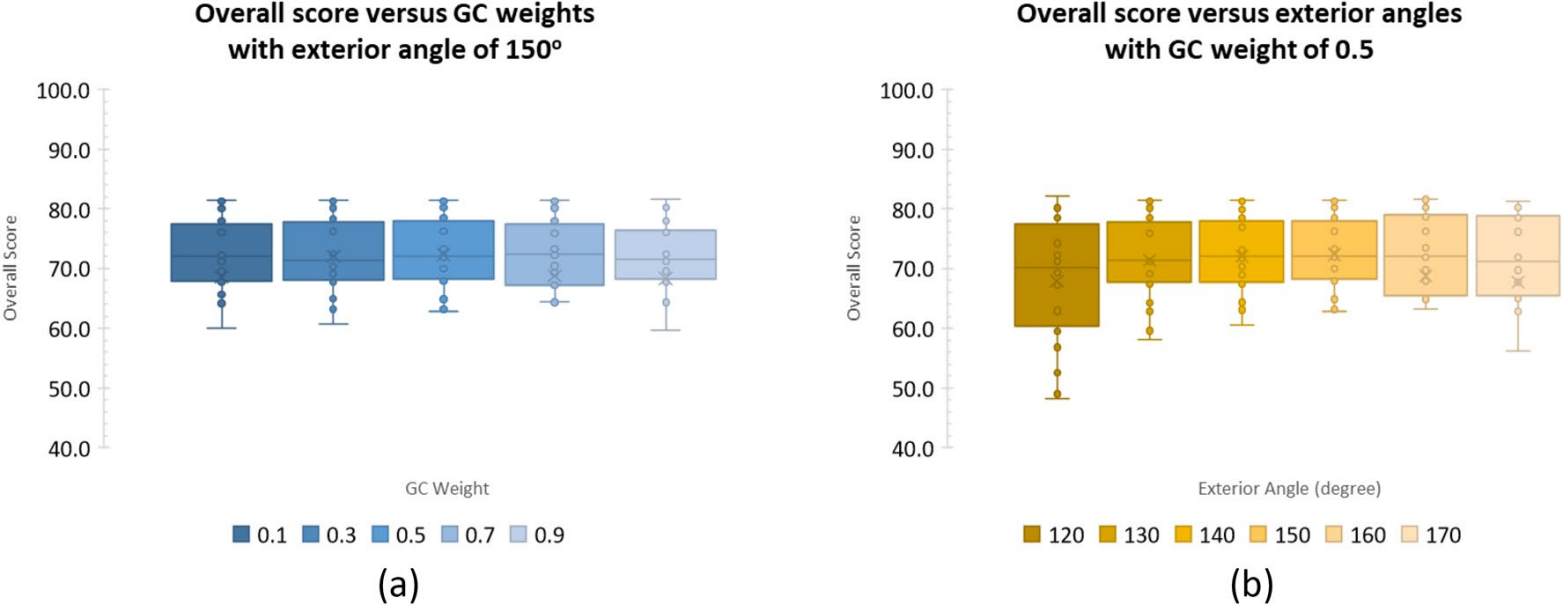
(**a**) Score variability with respect to Graph Cut weights, between 0.1–0.9. (**b**) Score variability with respect to Exterior angle, between 120–170 degrees.

Referred to these figures, the combination that yielded the highest overall score was chosen. As such, for the results reported herein, we set these numbers to 0.50 and 150°, respectively. Since these were the only empirical setups required, to assess the score variability due to these settings, Fig. 11 plots overall scores, when varying GC weights, with fixed exterior angles (a) and vice versa (b). It is evident that within optimal range, adjusting either of these parameters did not much affect the average scores, but slightly their deviations, in practice.

For numerical assessment, the proposed method (noted as *Proposed*) was benchmarked against those suggested by Liao^28^, Chen^12^, Yang^25^, Lu^4^, and Selver^17^, for the asymptomatic livers. Note that all metrics and processing time for^4,25^ and^17^ attributed to Liao^28^ implementation. While all 5 metrics provide insights into different aspects of segmentation, overall score computed by averaging corresponding scores are typically considered in comparing different methods^3^. Although the metrics and scores, presented in Table 4, were computed for each image, only average metrics (over all images) were listed in the referenced report^28^. Therefore, the scores, against which our method (denoted here as *Proposed*) was benchmarked and reported in Table 5, were computed from these averages (instead of the corresponding ones in each images), according to Table 3.

**Table 5:** Comparison evaluation metrics and score obtained by using different algorithms.

Method	VOE		RVD		ASD		RMSD		MDS	
	[%]	Score	[%]	Score	[mm]	Score	[mm]	Score	[mm]	Score
Liao^28^	5.8 ± 3.3	77.3	− 0.2 ± 4.0	98.9	1.1 ± 0.5	72.5	2.0 ± 1.3	72.2	21.6 ± 9.4	72.0
Chen^12^	6.5 ± 1.8	74.6	− 2.1 ± 2.3	88.8	1.0 ± 0.4	75.0	1.8 ± 1.0	75.0	20.5 ± 9.3	73.4
Yang^25^	8.9 ± 2.2	65.2	2.3 ± 2.0	87.8	1.4 ± 0.3	65.0	2.4 ± 1.2	66.7	24.3 ± 9.6	68.5
Lu^4^	7.4 ± 1.9	71.1	4.6 ± 2.8	75.5	1.2 ± 0.4	70.0	2.8 ± 1.3	61.1	38.5 ± 18	50.1
Selver^17^	11.9 ± 4.5	53.5	− 3.4 ± 5.2	81.9	1.7 ± 0.6	57.5	3.6 ± 1.8	50.0	49.3 ± 27.1	36.1
Proposed	*7.8* ± ***1.5***	*69.4*	*1.2* ± *2.1*	*93.6*	*1.3* ± ***0.3***	*68.5*	*2.6* ± ***0.7***	*64.5*	*24.2* ± ***6.6***	*68.6*

The italic means the values obtained by the proposed method.

The bold means the best values in each column.

**Figure 12:**
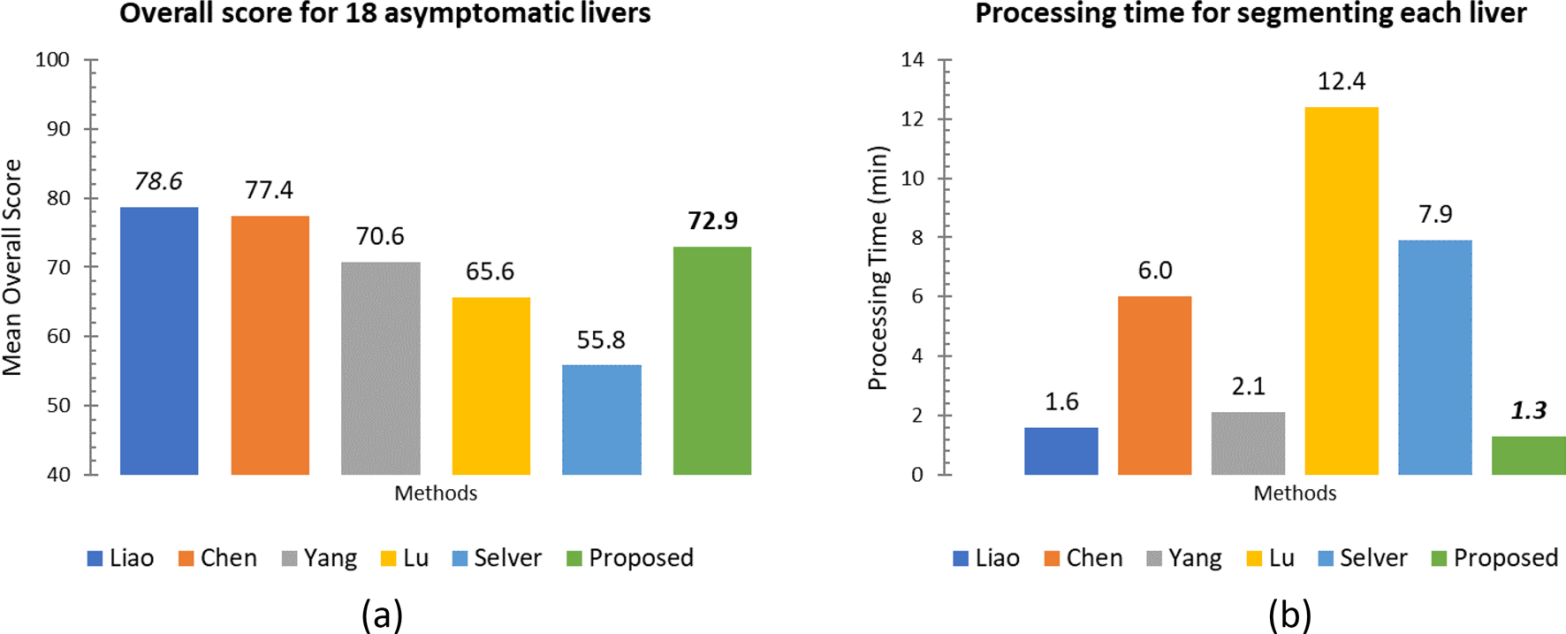
(**a**) Comparisons of the mean overall score among different methods. (**b**) Comparison of the mean processing time among different methods.

The mean overall scores and processing time are plotted in Fig. 12a and b, respectively.

Amongst these method, Liao’s work scored the highest in almost all measures, followed by Chen's and ours, respectively. It is worth pointed out that, both Liao’s^28^ and our methods worked best for the asymptomatic cases. However, neither the details of images considered nor individual metrics were reported therein. In addition, unlike ours, Cheng’s work required a dataset AAM for training. Compared to these state-of-the-art methods, the proposed one always ranked in top 2–4 in all evaluation metrics, i.e., VOE, RVD, ASD, RMDS, and MDS, with highest 2nd rank in RVD. It was ranked 3rd in overall score. Moreover, it did not involve pre-processing^28^ nor multiple landmarks being specified by a user^12^. Although the window/ level setting was not reported in^12,28^, it was fixed in our experiments to that for a Liver study. This setting might not be optimal in some instances, and thus led to low accuracy, as illustrated in Fig. 8. Nonetheless, thanks to intuitive constraints, the proposed method also required the least processing time. It took slightly faster than that proposed by Liao. This is because the latter had to build two models, i.e., intensity and PCA. Breaking down the process, our method took approximately 35 and 43 secs. to create the initial probability map and subsequent enhancements, respectively. It is also worth noting that, the standard deviation of all metrics, except RVD, evaluated on the proposed method was significantly lower than or equal to those of its counterparts. This implies that the proposed method produced consistent and reliable results, hence suitable for clinical practices.

In addition, segmentations on unlabeled (testing) dataset were also submitted to MICCAI website, for online evaluation. The resultant metrics and corresponding overall scores for 7 of 10 asymptomatic livers are presented in Table 6. Particularly, VOE, RVD, ASD, RMSD, MSD metrics were 8.0 ± 1.1, –0.3 ± 2.7, 1.3 ± 0.4, 2.5 ± 1.0, and 24.9 ± 10.0. These are hence converted to corresponding scores of 68.8, 88.3, 68.0, 64.5, and 67.1, respectively. Accordingly, the mean overall score was 71.3 ± 7.95. It is also noticed that, while the metrics varied across images, they were particularly low for case 08.

**Table 6:** The evaluation metrics and overall score obtained from 7 asymptomatic (unlabeled) cases.

CT image	VOE		RVD		ASD		RMSD		MSD		Overall score
	[%]	Score	[%]	Score	[mm]	Score	[mm]	Score	[mm]	Score	
01	7.4	71.1	2.4	87.1	1.1	71.9	2.0	71.9	19.7	**74.0**	75.2
02	8.4	67.2	− 2.1	88.4	1.2	71.2	2.2	69.2	21.2	72.1	73.6
05	9.6	*62.6*	0.9	**95.3**	1.5	60.3	2.7	61.6	24.2	68.0	69.6
06	8.6	66.4	3.0	83.8	1.3	66.7	2.2	69.2	20.7	72.7	71.8
07	6.6	**74.1**	0.3	98.2	0.9	76.6	1.8	**73.6**	23.3	69.2	**78.4**
08	8.6	66.5	− 4.5	*75.9*	1.9	*52.1*	4.7	*33.5*	47.2	*37.9*	*53.2*
09	6.8	73.3	− 2.0	89.3	0.9	**76.9**	1.9	72.8	18.2	76.0	77.7
Average	8.0	68.8	− 0.3	88.3	1.3	68.0	2.5	64.5	24.9	67.1	71.3
Std	1.1		2.7		0.4		1.0		10.0		

The bold means the best values in each column.

Similar to labelled dataset, the proposed method (noted as *Proposed*) was benchmarked* against those proposed by Peng^27^, Kainmüller^11^, Wu^20^, and Heimann^10^. The results are presented in Table 7. With greatest user’s intervention, Peng’s method outperformed the others in terms of all metrics. Meanwhile, statistical model employed by Kainmüller automatically took care of inter-subject variation, but took the longest to complete (15 min). In terms of processing time, Wu’s method was the fastest. However, automated ROI intiailization by MIP and thresholding was not reliable in presence of multiple or large lesions. The proposed method was the 2nd fastest, while being ranked 4th in terms of overall score almost identical to Wu’s, but much better RVD.

**Table 7:** Comparison evaluation metrics and score obtained by using different algorithms.

Method	Auto	Run Time (sec)	VOE [%]	RVD [%]	ASD [mm]	RMSD [mm]	MSD [mm]	Overall score
Peng^27^	*Semi*	120–180	**4.6** ± 0.5	1.0 ± 0.8	**0.7** ± 0.1	**1.5** ± 0.4	**16.9** ± 3.7	**83.4**
Kainmüller^11^	Yes	900	7.0 ± 2.3	− 3.6 ± 3.3	1.1 ± 0.3	2.3 ± 0.7	20.9 ± 6.4	73.0
Wu^20^	Yes	**27**	7.9 ± 1.3	1.3 ± 3.1	1.3 ± 0.2	2.5 ± 0.7	23.6 ± 8.1	71.4
Heimann^10^	Yes	600	11.0 ± 6.9	− 1.7 ± 8.4	2.4 ± 2.3	5.1 ± 4.9	35.2 ± 21.3	59.0
Proposed*	*Semi*	*78*	*8.0* ± *1.1*	− ***0.3*** ± *2.7*	*1.3* ± *0.4*	*2.5* ± *1.0*	*24.9* ± *10.0*	*71.3*

*Our scores were evaluated on 7 images, while other works were on 10 images.

The bold means the best values in each column.

As indicated in Table 8, the metrics obtained by the proposed method, when applying to both MICCAI labeled (training) and unlabeled (testing) datasets, were consistent.

**Table 8:** A comparison of evaluation metrics and overall score between labeled and unlabeled datasets.

Dataset	VOE		RVD		ASD		RMSD		MSD		Overall Score
	[%]	Std	[%]	std	[mm]	std	[mm]	std	[mm]	std	
Labelled	**7.8**	1.5	1.2	2.1	1.3	0.3	2.6	0.7	**24.4**	6.6	**72.3**
Unlabelled	8.0	1.1	− **0.3**	2.7	1.3	0.4	**2.5**	1.0	24.9	10.0	71.3

The bold means the best values in each column.

**Figure 13:**
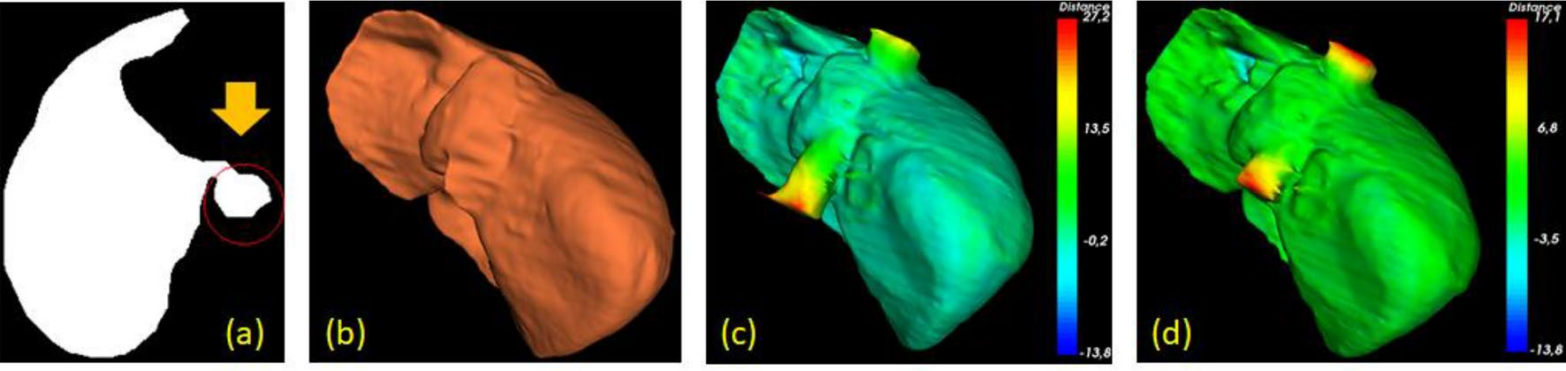
A case of a IVC being included in the segmented liver (**a**). The correct 3D ground truth is shown in (**b**). The reconstructed liver surfaces before (**c**) and after (**d**) manual IVC removal indicates significantly lower distance errors.

Visual and numerical assessments revealed one major pitfall of our method. Except errors, caused by ambiguous boundary between liver and other abdominal structures, which could only be elevated by means of statistically trained or deeply learnt models, the major cause of lower accuracies (compared to^12,28^) was due to inferior vena cava (IVC). It was cylindrical and appeared oval in a cross-sectional image that connects to the main branches of hepatic vein. But it was not considered as a part of the liver, hence excluded from the ground references. Nonetheless, it is anticipated that including IVC in surface reconstruction did not make a low-quality 3D model, especially in pre-operative planning. If it were, however, really necessary to remove this structure, a contrast agent enhancing blood passage, could be administered. Alternatively, a model-based approach, targeting a tubal structure, could be employed. To confirm the hypothesis, we manually removed portions of this IVC in one dataset and found that the overall score increased from about 72 to 79. To this end, a user could choose few slices above and below the liver, where vena cava is found. Excluding these slices would effectively disconnect it from the liver. Besides, adding this step would not cause much burden to the user, in addition to specifying a seed point. That being said, it would increase observer variability, and hence was not included in the above analyses. Figure 13 illustrates the segmented liver before and after partial removal of IVC at bottom.

**Figure 14:**
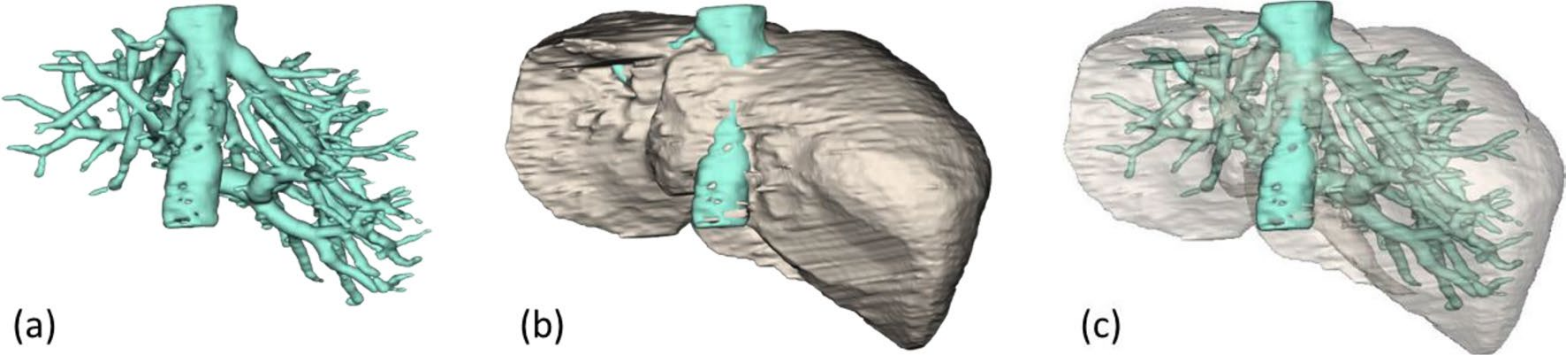
Simultaneous extraction of a liver (**b**) and its vasculature, including IVC (**a**), with their fusion (**c**).

Alternatively, the liver and entire hepatic vasculature could be independently segmented. To this end, parts of the proposed method could be exploited. Particularly, MND and RL, without GC or related constraints, were simultaneously applied to extract interior vessels, which were subtracted from and later fused with the liver. The extracted result is illustrated in Fig. 14. This vessel segmentation process took about additional 1.2 min.

With the proposed anatomical constraints, our method was specifically designed for segmenting a healthy liver^4,17,28^. Consequently, it did not work well in highly pathological cases, especially when lesions, with similar intensity to the background, are present on liver boundary. To demonstrate the limitation of current study, further evaluations were performed on such cases in labelled dataset, where moderate size (image 10) and large (image 16) tumors were on the boundary of the liver. The resultant metrics were reported in Table 9.

**Table 9:** The results of segmentation on cases with extensive tumors.

CT Image	VOE		RVD		ASD		RMSD		MSD		Overall score
	[%]	Score	[%]	Score	[mm]	Score	[mm]	Score	[mm]	score	
10	9.4	63.5	1.0	94.5	1.7	58.0	3.7	48.6	34.9	54.8	63.9
16	23.2	9.6	− 13.1	30.1	3.8	5.1	8.5	0	55.8	27.7	14.5
Average	16.3	36.6	− 6.1	62.3	2.8	31.6	6.1	24.3	45.4	41.3	39.2

**Figure 15:**
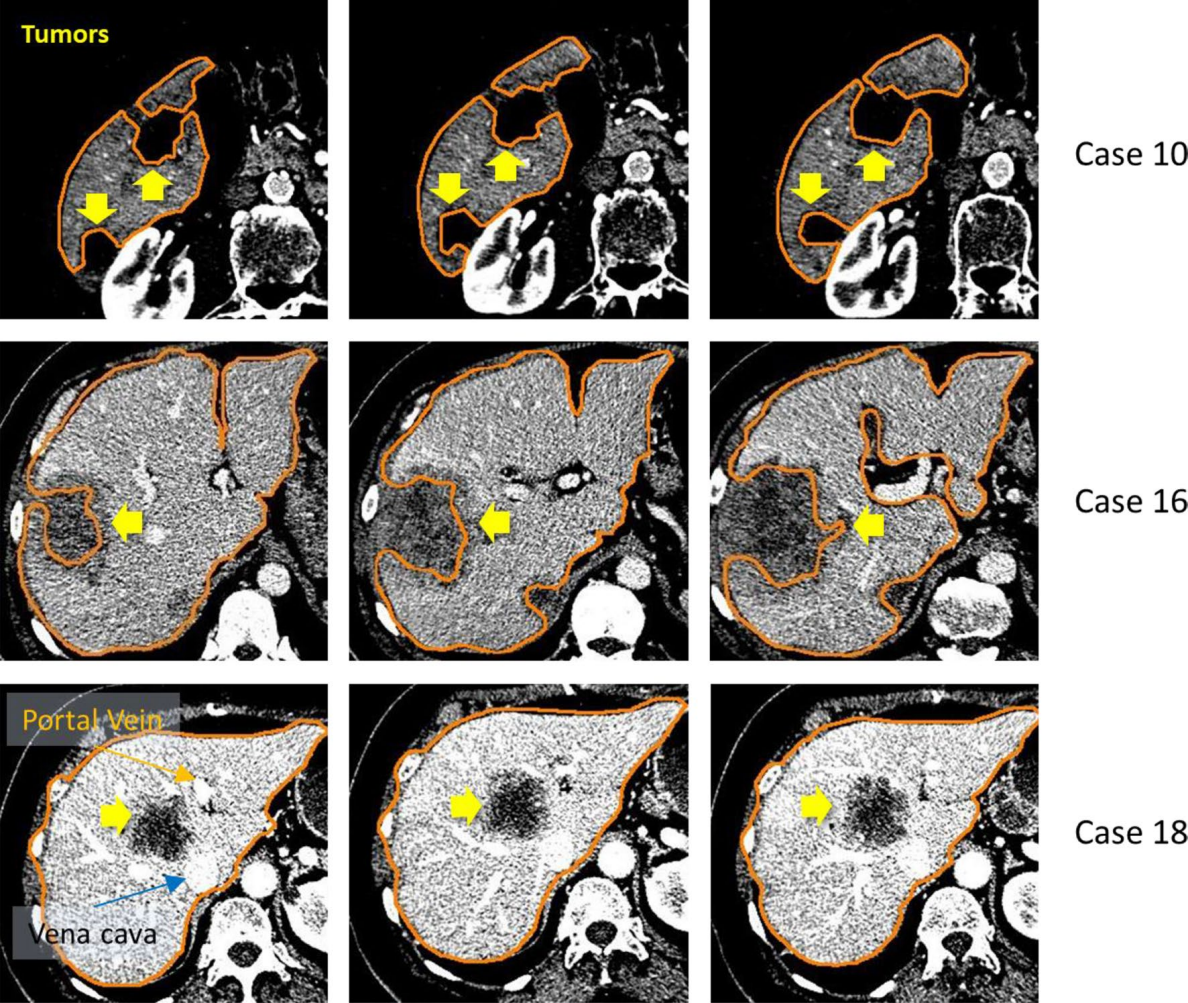
Segmentations of diseased livers. For the case number 18 (included in Table 4 but not in Table 9), the lesion was relatively small and located inside the liver, near vena cava and portal vein entry.

Figure 15 illustrated segmented mildly (18) and severely (10 and 16) pathological livers on selected slices. Note that, despite interior voids, delineated liver in case 18 is valid and hence reported in Table 4.

While the liver in case 18 was successfully segmented, the other cases were not. This was due to the healthy parts enclosing the lesion (located near vena cava and portal vein entry) remained valid, according to the anatomical constraints. There exist several methods specifically developed for tumor delineation and can be integrated into our scheme during post-processing. Their detailed analyses and treatments, however, fell out of scope of this study and thus left for future investigation.

## Conclusion

In this paper, a novel scheme for semi-automatic liver segmentation was presented. It was based on an MND of pixel statistics of low orders, constructed from a small patch surrounding a seed point. Unlike other intensity based MND, the proposed multi-dimensional RV suppressed imaging noise, while discriminated liver ROI against neighboring background. Once the corresponding probabilistic map was created. The liver was then automatically segmented by enhancing and optimizing a network of spatio-intensity contexts, by using RL and GC methods, respectively. A straightforward yet intuitive heuristic anatomical conditions of a normal liver were subsequently imposed on the segmented shape, by using BN detection and adjacent contour constraints, during the post-processing. The developed system was implemented on a typical computing system with moderate resources and validated on both labeled and unlabeled volumetric CT images, obtained from MICCAI SLIVER07 database. There were, in total, 25 and 2 asymptomatic and symptomatic cases, respectively, analyzed in the above experiments.

Unlike many existing works, the proposed method did not require much expertise on the liver anatomy, but only one interior point representing general appearance to begin with. Furthermore, it did not require model training nor any final adjustment (unless in pathological instances). Benchmarked against most recent works, the proposed method scored 3rd on labeled dataset and 4th on unlabeled one. In particular, it did best on RVD (i.e., false positive rate), whose score was seconded to only that using a statistically trained model. Moreover, in terms of processing time, it was fastest in its class. The entire process took only 1.3 min to segment a whole 3D liver, thanks to trivial anatomical and geometrical assertions. Lastly, our method performed consistently well on both labelled and unlabeled datasets.

Thus far, there remained drawbacks associated with the proposed method. With preset intensity range, it failed to accurately separate, for example, the liver from an adjacent organ, in low contrast samples. Since processing time was relatively short, interactively adjusting these viewing parameters during segmentation could help improving accuracy in those cases. Lastly but more importantly, the method was not specifically devised for a liver with overwhelming inhomogeneity. As such, it failed to separate liver from tumors or embedded vessels with similarly intensities. Nonetheless, we have shown that simultaneously extracting a liver and its vasculature, could help resolving this issue. It has also been demonstrated elsewhere that tumors may be explicitly delineated prior to being subtracted from the liver.

Therefore, further improvements should be considered in future work. These include issues on disentangling connective parts, e.g., IVC, portal vein, and kidney, etc. Moreover, the proposed method produced satisfactory results only in healthy livers and those with minor lesions. Therefore, investigation into incorporating tumor extraction is vital before it could be readily applied in screening CAD system. Extracting tumors, while performing liver segmentation is nontrivial and remains challenging research area. Some studies addressed this issue by filling voids (or lesions) by morphological operator^22^, regulating implausibly deformed shape by means of statistical model^13^, coupled extraction of both liver and tumor by texture^18^ or deeply trained models^16^. Others opted more straightforward level set^25^ evolution or region growing^26^, on tumor ROI.

On automating the process, a seed point can be determined from imaging information^19,20^, enabling fully unsupervised analysis. That way, several points can be initialized simultaneously^12^, to capture a wider range of pixel inhomogeneity and hence much reliable model. In addition to statistical variables, gathered from pixel intensities, their texture features can be incorporated into this multivariate distribution, to also enhance both appearance and shape descriptions of a liver. Another future direction, worth considered, is extending the RL and GC into the 3rd dimension, taking into account interslice spatial continuity.

It may be concluded from the experiments and analyses that the proposed method could extract healthy livers as well as those mildly diseased. Meanwhile, it failed to segment livers with extensive lesions and those severely deformed due to one. Nevertheless, extracting a normal or a virtually normal liver could help physicians, for examples, to better visualize and determine post-surgery or post-treatment, and their prognostic outcomes, etc.^43^ Moreover, in liver transplant, the proposed method can be applied to living donor, whose tumor or other pathological conditions is not anticipated. In fact, it has been shown elsewhere^44,45^ that extracting a liver is a precursor toward virtual liver resection in preoperative planning for transplantation. Accurately localizing functional sub-segments of a liver, their volumes, and vasculature, are considered vital for ensuring liver regeneration and hence reducing risk of post-operative liver failure.

## Data Availability

Volumetric CT images of livers used in this study was obtained from SLIVER07 dataset. It was available at http://www.sliver07.org/.
